# Structural models of intrinsically disordered and calcium-bound folded states of a protein adapted for secretion

**DOI:** 10.1038/srep14223

**Published:** 2015-09-16

**Authors:** Darragh P. O’Brien, Belen Hernandez, Dominique Durand, Véronique Hourdel, Ana-Cristina Sotomayor-Pérez, Patrice Vachette, Mahmoud Ghomi, Julia Chamot-Rooke, Daniel Ladant, Sébastien Brier, Alexandre Chenal

**Affiliations:** 1 Institut Pasteur, UMR CNRS 3528, Chemistry and Structural Biology Department, 75724 PARIS cedex 15, France; 2 Sorbonne Paris Cité, Université Paris 13, Groupe de Biophysique Moléculaire, UFR Santé-Médecine-Biologie Humaine, 74 rue Marcel Cachin, 93017 Bobigny Cedex, France; 3 Institut de Biologie Intégrative de la Cellule, UMR 9198, Université Paris-Sud, F-91405 ORSAY Cedex, France

## Abstract

Many Gram-negative bacteria use Type I secretion systems, T1SS, to secrete virulence factors that contain calcium-binding Repeat-in-ToXin (RTX) motifs. Here, we present structural models of an RTX protein, RD, in both its intrinsically disordered calcium-free Apo-state and its folded calcium-bound Holo-state. Apo-RD behaves as a disordered polymer chain comprising several statistical elements that exhibit local rigidity with residual secondary structure. Holo-RD is a folded multi-domain protein with an anisometric shape. RTX motifs thus appear remarkably adapted to the structural and mechanistic constraints of the secretion process. In the low calcium environment of the bacterial cytosol, Apo-RD is an elongated disordered coil appropriately sized for transport through the narrow secretion machinery. The progressive folding of Holo-RD in the extracellular calcium-rich environment as it emerges form the T1SS may then favor its unidirectional export through the secretory channel. This process is relevant for hundreds of bacterial species producing virulent RTX proteins.

Disorder-to-order transitions play a key role in the biological functions of many proteins that contain intrinsically disordered regions^1^,^2^. Structural disorder predictors estimate that bacterial, viral and eukaryotic proteins contain large disordered regions, mainly involved in cell signaling, physiological and patho-physiological processes, including cancer and neurodegenerative diseases. In many instances, intrinsically disordered proteins adopt a defined three-dimensional structure after binding to ligands, and thus serve as “molecular switches” to control the biological functions of the corresponding proteins and/or ligands. We recently characterized a novel class of intrinsically disordered polypeptides that are constituted by so-called Repeat-in-ToXin (RTX) motifs found in many important virulence factors, widely distributed among Gram-negative bacterial species^3^,^4^. RTX motifs are calcium-binding, nona-peptide sequences, that are repeated in a tandem fashion (from 6 to more than 50) and fold in the presence of calcium into a parallel β-roll structure^5^. Although RTX-containing proteins display a variety of biological functions, they all require calcium binding to carry out their functions and they are all secreted by a type 1 secretion system (T1SS)^6^,^7^,^8^. The fact that most RTX proteins are secreted by the T1SS suggests that the RTX motifs are key structural features to favor efficient secretion through the T1SS pathway.

One of the best-characterized members of the RTX family is the adenylate cyclase (CyaA) toxin from *Bordetella pertussis*, the causative agent of whooping cough. CyaA is able to invade eukaryotic cells where it is activated by calmodulin (CaM) to produce supraphysiological cAMP concentrations that are toxic for the target host cells. CyaA is a 1706-residue long protein organized in a modular fashion (See Figure S1)^9^,^10^ with a N-terminal catalytic domain (ACD: adenylate cyclase catalytic domain, residues 1–400^11^,^12^, a translocation region (TR, residues 400–500)^13^,^14^, a hydrophobic region (HR, residues 500–750, an acylated region (AR, residues 850–1000)^10^,^15^,^16^, and a C-terminal RTX domain (RD, residues 1006–1706) involved in binding to the specific cell receptor CD11b/CD18. Upon interaction of RD with the cell surface receptor αMβ2 integrin, the N-terminal cyclase domain translocates into the host cell cytosol where it generates supraphysiological cAMP concentrations, which inhibit host cell anti-bacterial activities.

RD comprises ~40 copies of RTX motifs, organized in five successive blocks (named I to V) of 8–10 RTX motifs, separated by non-RTX flanking regions of variable length (23 to 49 residues)^17^. RTX motifs constitute the main Ca^2+^ binding sites of the protein^18^ and consequently, are directly implicated in the calcium-dependent cytotoxic activities of the toxin, *i.e.*, cell receptor-binding, ACD translocation and plasma membrane permeabilization^16^,^19^. Hence, the elucidation of the structure-to-function relationship of calcium binding to RD is essential in understanding the mechanism of calcium-dependent regulation of the biological functions of CyaA and more broadly-speaking, of the RTX toxins themselves^20^. We have previously shown that RTX proteins, such as RD^21^,^22^ and isolated blocks thereof^19^,^23^,^24^, exhibit the hallmarks of intrinsically disordered proteins in the absence of calcium (*i.e.*, Apo-form); they adopt disordered conformations mainly due to electrostatic repulsions between negatively charged residues^25^. Calcium binding triggers a strong reduction of the mean net charge, dehydration, compaction, folding and stabilization of secondary and tertiary structures of RTX proteins^26^.

Here, we present structural models for both the Apo- and the Holo-forms of RD by combining Small Angle X-ray Scattering (SAXS), Raman spectroscopy and Hydrogen/Deuterium eXchange-Mass Spectrometry (HDX-MS) data. We show that the molecular dimensions of RD support the hypothesis that the ensemble of intrinsically disordered conformations of the Apo-form in the low calcium environment of the bacterial cytosol facilitates RD secretion through the narrow channel of the bacterial T1SS. Conversely, upon reaching the extracellular, calcium-rich medium, RD folds into a compact, stable and functional structure whose molecular dimensions may prevent any backtracking into the secretion channel, thus favoring the overall secretion of the protein.

## Results

### Structural parameters and model-free analysis of RD from SAXS data

**Figure 1:**
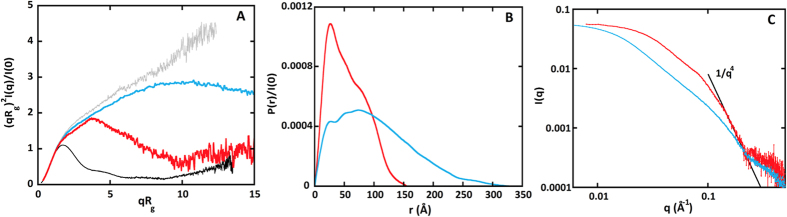
SAXS patterns of Apo- and Holo-RD. (**Panel A**): Dimensionless Kratky representations of Apo-RD (cyan line) and Holo-RD (red line) SAXS patterns, together with the SAXS curve of IB5 (grey line), a small fully unfolded proline-rich salivary protein^30^ and that of PolX (black line), a fully structured, globular protein^31^. (**Panel B**): P(r) distance distribution functions of Apo-RD and Holo-RD. Same color code as in panel A. (**Panel C**): log-log representation of Apo-RD and Holo-RD SAXS patterns. The same color code is used as in panel A.

SAXS is an instrumental approach for the characterization of intrinsically disordered proteins and ligand-induced conformational changes^27^,^28^. Scattering patterns of Apo-RD and Holo-RD (RD, residues 1006 to 1706 of CyaA, see Supplementary Information for experimental procedures) were recorded using the on-line Size Exclusion-High Performance Liquid Chromatography (SE-HPLC) column available at the SWING beamline of the SOLEIL Synchrotron. The values of the radius of gyration, the maximum diameter and the molecular mass derived from the scattering curves of monomeric Apo-RD and Holo-RD are presented in Table S1. The scattering curves are shown in Fig. 1A using a dimensionless Kratky representation^29^, together with those of IB5, a fully unstructured proline-rich small salivary protein^30^ and PolX, a fully structured, closed-to-spherical protein^31^, as a matter of comparison. The distance distribution curves are shown in Fig. 1B. It is immediately apparent from the R_g_ reduction, P(r) and Kratky profiles that Apo-RD is essentially unstructured and undergoes a major transition to a much more compact conformation upon calcium binding. Indeed, the extremely high values of both R_g_ and D_max_ for Apo-RD (84 Å and 330 Å, respectively) are not compatible with a compact, folded structure of a protein of 72 kDa. The R_g_ and D_max_ values of Holo-RD (44 Å and 155 Å, respectively) are also larger than those expected for a globular protein of the same molecular mass (*ca* 27 Å and 65 Å, respectively). The Kratky plot of Apo-RD has a similar shape to that of fully unstructured IB5 protein up to qR_g_ ≈ 7, before leveling off at larger angles. This suggests the existence of residual elements of local structure. Conversely, the Holo-RD curve reaches a maximum, followed by a noticeable decrease. This is indicative of a well-folded, compact object, for which a clear interface with the solvent can be defined. The position of the maximum at a larger qR_g_ value than that of the PolX curve (where a shoulder is visible on the Holo-RD curve) is associated with a markedly anisometric shape of the protein. These interpretations are further supported by the analysis of the two scattering curves plotted in log-log representation in Fig. 1C. Noticeably, the curve of Holo-RD exhibits a region with a q^−4^ dependence characteristic of the presence of a sharp interface between the solvent and a compact, well-folded domain, as stated in Porod’s law^32^. This indicates that a significant part of the molecule is well structured, in contrast with the curve of Apo-RD where no q^−4^ slope can be observed. The conclusion of this model-independent analysis of the two scattering curves is that Apo-RD behaves as an unstructured polymeric chain with no structured globular regions, but with local, transient structural elements, while in the presence of calcium, Holo-RD adopts a compact, folded and anisometric conformation.

### Apo-RD modeling as a statistical polymer chain

In the absence of complementary information on the presence and possible localization of secondary structure elements (obtained, for instance, by Nuclear Magnetic Resonance(NMR)), no detailed structural models can be meaningfully elaborated from the SAXS data. In contrast, a relevant description is provided by the theory of statistical polymer chains. If we consider the protein as a statistical polymer chain with a persistence length, the scattered intensity I(q) is given by the expression of Sharp and Bloomfield^33^:


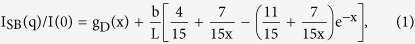


where b is the length of the statistical element, twice the persistence length of the chain, L is the contour length of the chain, x is a dimensionless quantity x = q^2^Lb/6 and g_D_(x) is the Debye function 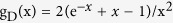. Finally, we must take into account the thickness of the chain through the value of the radius of gyration of cross section R_c_ that modifies the expression of the scattered intensity as follows:


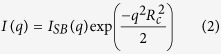


The two parameters L and R_c_ are related by the volume of the protein V = 2πR_c_^2^L = v_p_M/N_a_, where N_a_ is the Avogadro number, M the molar mass of RD (72,624 Da) and v_p_ the partial specific volume with a value of 0.713 cm^3^/g, estimated from the protein amino acid sequence. The model is thus entirely defined by two independent parameters, b and L.

**Figure 2:**
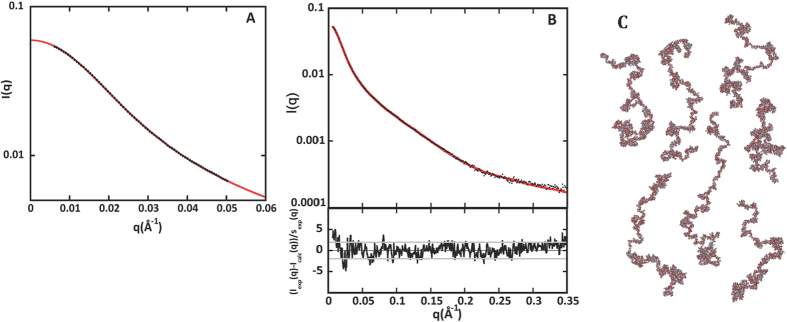
Modeling of Apo-RD. (**Panel A**): Fit of the smaller-angle region (qb<3) of the experimental scattering data of Apo-RD (black dots) using equation 2 (red line); (**panel B**): Fit of the Apo-RD SAXS pattern (black dots) using EOM. The fit (red line) is the average scattering pattern of the seven conformations shown in (**panel C**). In (**panel B**) the bottom frame presents the corresponding distribution of reduced residuals.

The fit to the experimental data of Apo-RD is excellent in the region of validity of the expression of equation (2) (qb < 3) as shown in Fig. 2A. The corresponding values of L, b and the derived value of R_c_ are presented in Table S1. Thus the scattering properties of Apo-RD at small-angle are that of a polymer chain comprising around 10 statistical elements of an approximate length of 80 Å and an approximate diameter of 13 Å. With a value close to 700 Å, L is significantly lower than the contour length of a fully unfolded 701 residue long polypeptide chain (that should be in the order of 2500 Å) while b (about 80 Å) has a much larger value than that generally accepted for an unstructured polypeptide chain (in the order of 18 Å). Both values point towards the presence of a significant amount of residual structural elements in Apo-RD.

### Apo-RD modeling using conformational ensembles

The description of the protein given above yields statistical average values for several parameters over the population of molecules. We tried to illustrate this distribution of conformations using the ensemble optimization method (EOM). We used the three different types of reconstructed linkers offered by the EOM program (see Materials and Methods for details). As shown in Fig. 2B, a good fit with our experimental intensities (χ = 1.40) was obtained when using the “compact” reconstructed linkers, while, strikingly, the use of “random” and “native” linkers gave only very poor fits. Figure 2C presents the corresponding ensemble of seven conformations, the average calculated scattering curve of which is shown by the red line in panel B. Running the genetic algorithm in the GAJOE program multiple times yields different ensembles of conformations that should be therefore considered as illustrations of the polypeptide chain main features, rather than actual conformations adopted by the protein. Locally folded regions are apparent, in agreement with the dimensionless Kratky representation, with the absence of q^−4^ behavior and the modeling of Apo-RD as a statistical polymer chain that exhibits local structural elements.

### Holo-RD *ab initio* modeling from SAXS data

**Figure 3:**
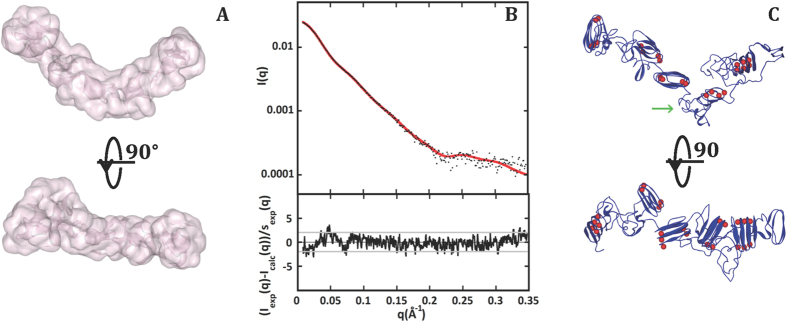
Modeling of Holo-RD. (**Panel A**): Two orthogonal views of a typical *ab initio* model of Holo-RD obtained by the program Gasbor; (**panel B**): two orthogonal views of a typical model of Holo-RD obtained using the program Bunch and the models proposed by Phyre2 for six individual regions of the protein, as explained in Materials and Methods; the green arrow points towards the central region between Blocks III and IV; (**panel C**): the calculated scattering pattern of the Bunch model shown in panel B (red line), superimposed onto the Holo-RD experimental SAXS data (black dots); the bottom frame presents the corresponding distribution of reduced residuals.

Structural parameter values and the dimensionless Kratky plot indicate that in the presence of calcium, RD adopts monomeric and compact conformations. Yet, comparison of the D_max_ value of 155 Å to the diameter of a spherical protein of the same molecular mass (*ca* 70 Å) indicates that Holo-RD exhibits an elongated shape. We determined potential protein envelopes by using the Gasbor program^34^. Out of 100 runs, 25 yielded a χ−value equal to or lower than 1. These computed conformations had very similar characteristics, with an average normalized spatial discrepancy (NSD) value of 1.35^35^. One typical conformation, similar to a curved elongated cylinder, is shown in Fig. 3A.

### Calcium-binding induces an increase of β-sheet content at the expense of structural disorder

**Figure 4:**
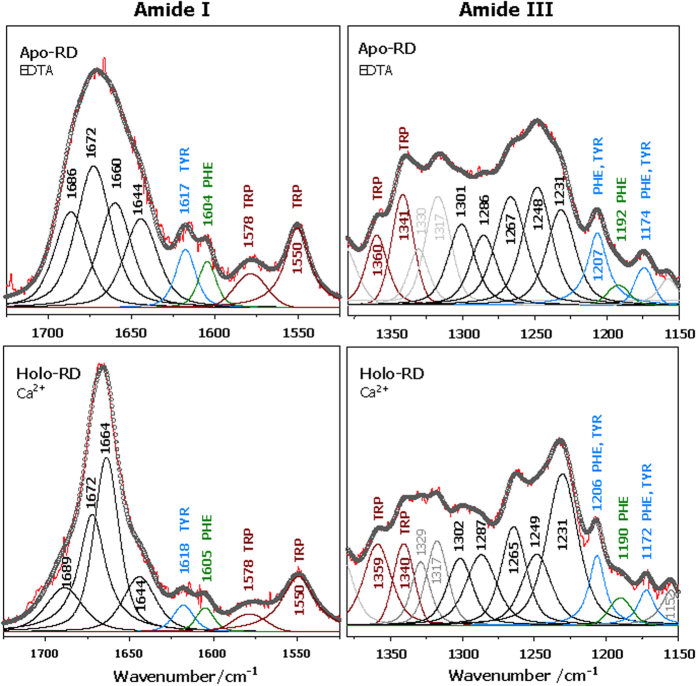
Raman spectroscopy of RD. Experimental Stokes Raman spectra of Apo-RD (top) and Holo-RD (bottom) show Amide I (left) and Amide III (right) regions used for secondary structure determination, as well as characteristic bands from aromatic amino acid side chains. Experimental spectra (red color) and decomposition of the amide I (left) and amide III (right) regions of Raman spectra are shown: traces representing different structural elements are in black color, those from amino-acid side chains are indicated in specific colors (F in green, Y in blue, W in red); circles correspond to the fitted profiles (sum of components). See Table S2 for details.

The profile analysis of amide I (1700–1600 cm–1) and amide III (~1320–1220 cm–1) regions acquired by Raman spectroscopy provides an estimation of secondary structure content (see Table S2, Figures S2 and S3). Data obtained at 20 °C shows that the secondary structure of Apo-RD comprises 25% of random coil, 55% of turns and 20% of beta-sheet (Fig. 4 and Table S2). In the presence of 4 mM calcium, the beta-sheet content increases from 20 to 37% at the expense of structural disorder, which decreases down to 14% in the Holo-state. The global turn content decreases slightly (6%) and structural differences in turn shape (turn type) are also evidenced. Similar experiments were performed in the absence of calcium at 40 and 50 °C, temperatures at which RD only populates the unfolded Apo-state^21^,^22^. In these conditions, the beta-sheet structures disappear (Figure S3 and Table S3), and Apo-RD is mainly composed of turns (70%) and random coils (30%). In the Holo-state, the secondary structure content does not change from 20 to 50 °C, in agreement with the previously observed temperature stability of Holo-RD^21^,^22^. Figure S4 shows the regions containing characteristic contributions from aromatic side chains F, Y, W (see Supplementary Information for details on Raman spectra processing). The aromatic residues are mainly solvent-exposed in the Apo-state (Table S4), while in the Holo-state, they sample hydrophobic environments, as suggested by changes in intensity and position of characteristic Raman bands.

### Residual secondary structure is primarily found in the C-terminal flanking region of Apo-RD

HDX-MS experiments were carried out to probe the structural features of RD in both its Apo and Holo-states. The rate of amide hydrogen exchange is influenced by both the solvent accessibility and the structure of the protein. Amide hydrogens fully exposed to the solvent and not involved in structural elements exchange rapidly, whereas those found within secondary and tertiary structures exchange more slowly due to hydrogen bonding^36^,^37^. Amide hydrogens can therefore be used as a probe to distinguish intrinsically disordered regions from those containing secondary or tertiary structures^38^,^39^.

**Figure 5:**
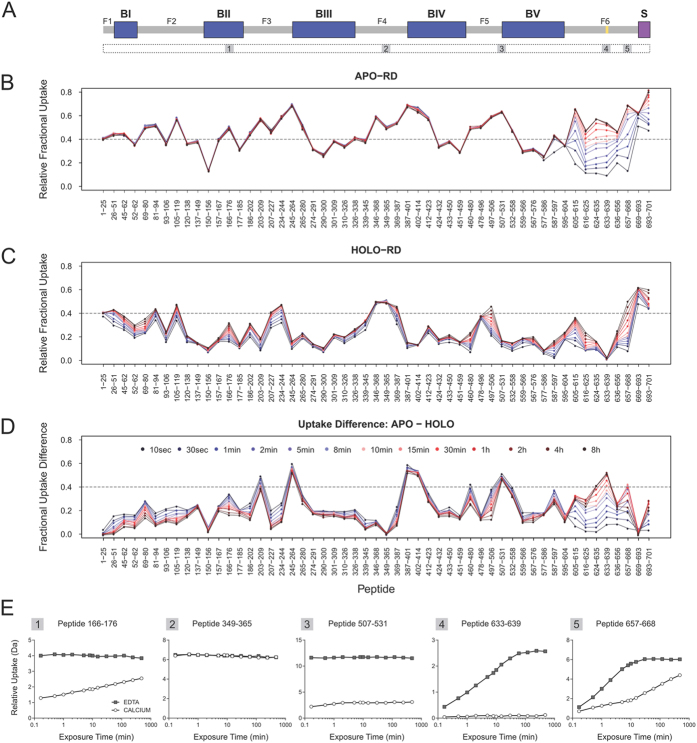
Exchange behavior of the RD protein in Apo- and Holo-states. (**A**) Schematic representation of the RD protein showing the position of the five blocks containing RTX motifs (BI to BV; colored in blue) and of the flanking regions (F1 to F5; colored in gray). The F6 region is highlighted in red, the Block A in yellow, and the secretion signal (S) in purple. The positions of the five peptides shown in panel D are displayed below this scheme. (**B**,**C**) Profiles of RD in both the Apo- and Holo-state. The relative fractional exchange data calculated at each time point was plotted as a function of peptide position. Relative fractional exchange values were determined as described in the experimental section. The red to blue lines correspond to data acquired form 10 sec to 8 h deuteration, respectively (see color legend in panel D). Each dot corresponds to an average of three independent HDX-MS experiments. (**D**) Fractional uptake difference plot showing the differences in uptake calculated between Apo- and Holo-RD at each time point, and for each peptide. A high uptake difference value indicates a calcium-induced protective effect, while a low value is indicative of a weak effect of calcium binding. (E) Deuterium uptake curves for selected peptides in the Apo- (filled squares) and the Holo- (open circles) states. Each example illustrates a unique HDX behavior, as monitored during the time course of our experiment.

To investigate the structural organization of Apo-RD, the protein was digested with pepsin and 53 peptides covering 98.1% of the protein sequence were selected for HDX data analysis (Figure S5). The kinetics of deuterium exchange was analyzed for all regions of RD, including Blocks I–V, the flanking regions separating each RTX Block (annotated F1 to F6 in Fig. 5A) and the secretion signal (S). The relative fractional exchange of three independent replicates was calculated for each peptide at each time point from 10 s to 8 h and plotted as a function of peptide position (Fig. 5B,C). This type of representation was employed to easily visualize and compare the relative deuterium incorporation across the entire protein sequence, providing both spatial and temporal information.

In the Apo-form of the protein, dynamic events were only observed in several C-terminal regions, and more particularly in the C-terminal flanking region F6, where significant increases in the rate of deuteration over the time course of the experiment were observed. This feature is characteristic of the presence of secondary structural elements (Fig. 5B). In contrast, no dynamic events were observed in the upstream part of the protein, *i.e.*, from residues 1 to 586, where the maximum level of deuteration was immediately reached (within 10 s), as observed in intrinsically disordered regions fully exposed to the solvent^40^. However, differences of solvent accessibility, *i.e.*, a heterogeneous pattern of deuterium uptake, were noticed within this largely exposed region. The highest levels of deuteration were primarily located in the N-terminal part, or immediately upstream, of each RTX block (*e.g.*, residues 105–119 in F2, 234–264 in F3, 387–423 in Block IV, and 497–531 in Block V). Conversely, regions with less exchangeable amide hydrogens are located within the C-terminal part of RTX blocks: residues 52–62 in Block I, 150–156 and 177–185 in Block II, 274–345 in Block III, 424–459 in Block IV, and 559–586 in Block V. A noticeable difference in accessibility was also globally observed between RTX blocks, with Blocks I and II more solvent-exposed than Blocks III to V. The flanking regions F1 to F5 had high levels of deuterium uptake and are thus expected to be largely solvent-exposed (Fig. 5B).

### HDX-MS highlights dramatic changes in the structure and dynamics of Holo-RD

Calcium-induced conformational rearrangements of RD were initially characterized by monitoring the exchange rate of RD in both the presence and absence of calcium at the intact protein level (Figure S6). Calcium binding induced a massive reduction in the overall deuteration of the protein, indicating a dramatic change in the global structure and dynamics of the protein in the two states. The drastic structural changes between the Apo and the Holo forms were confirmed upon analysis at the peptide level. The overall deuteration pattern of Holo-RD was greatly decreased as compared to that of Apo-RD all along the polypeptide chain (Fig. 5C). Dynamic processes, revealing the presence of structure, were primarily located in those areas within Block I and those flanking RTX Blocks II-V. More specifically, dynamic events occurred between residues 26–119, 166–176, 186–244, 369–387, 460–480, and 497–506. Furthermore, the C-terminal F6 region displayed dynamic events from residues 605–632 and 640–669, but was much less dynamic compared to that observed in the Apo-state. Strikingly, a short segment, encompassing residues 631–645 in the middle of the F6 region, demonstrated essentially no observable dynamic processes (as judged by peptide 633–639) indicating a nearly complete protection from the solvent. This segment corresponds to the so-called “Block A” region (residues 631–645) that is conserved among various RTX proteins, and was shown to be essential for folding of the CyaA Block V polypeptides^17^,^19^,^23^. This site may indeed serve as the nucleating core for the Block V and subsequently, the entire RD protein (see Supplementary Discussion).

Figure 5D plots the difference in relative fractional uptake between Apo- and Holo-states, which provides a more quantitative assessment of the differences between the two states. A high uptake difference value indicates a calcium-induced protective effect on the exchangeable amide hydrogens, while a low value is indicative of a weak effect of calcium binding. With the exception of Block I and Block II, the greatest effects induced by calcium were mainly detected at the N-terminus of Block III to Block V, at the N-terminus of flanking regions F3 and F5, and in Block A of the F6 region. A massive reduction in solvent accessibility was observed in the calcium-binding RTX sequences. The highest (>76–100%) levels of protection were centered on the RTX domains, from residues 245–264 (Block III), 387–414 (Block IV), and 507–558 (Block V). Intermediate (>25%) levels were located at residues 137–149, 157–185, 265–345, 412–459, 559–586, also situated within RTX Blocks, and 605–615 of the F6 region. The strong reduction in the number of labile amide hydrogens available for exchange is in excellent agreement with the calcium-induced dehydration, compaction and folding of RTX motifs previously documented^26^. These calcium-induced changes are summarized in Figure S7. Interestingly, a slight protection from the solvent was observed in Block I and dynamic events were only observed in this region, suggesting that this Block is folded and solvent-exposed. Finally, three regions of RD, residues 1–25, 349–365 and 669–693, demonstrated neither dynamic events nor conformational change upon calcium binding.

Deuterium uptake curves for selected peptides in the Apo- and Holo-states are plotted in Fig. 5E to illustrate the diversity of conformational states. Each selected peptide corresponds to a unique HDX behavior, as monitored during the time course of our experiment. The region of peptide 1 (residues 166–176 in Block II) is intrinsically disordered in the Apo-state, and acquires structure in the Holo-state. Peptide 2 (residues 349–365 in F4) is intrinsically disordered, devoid of structure in both Apo- and Holo-states, with no observable effect of calcium addition. Peptide 3 (residues 507–531 at N-terminus of Block V) exhibits the same behavior as peptides 1 and 2 in the Apo-state, but a strong masking effect is observed in the Holo-RD form. Peptides 4 (residues 633–639 of Block A) and 5 (residues 657–668 of the F6 region) both display the presence of secondary structure elements in the Apo-state. Binding of calcium induces a complete masking effect for peptide 4, suggesting that this region is fully buried in the folded structure of RD, as discussed above (see also Supplementary discussion for more details), whereas a change of dynamics was observed for peptide 5, indicating that this latter is more solvent-exposed than the former. All these HDX behaviors are also reported in Figure S7, which schematically highlights the diversity of calcium-induced changes.

### Molecular model of Holo-RD

We attempted to combine our SAXS, Raman and HDX-MS data to propose a putative molecular model of Holo-RD in solution. In the presence of calcium, the tandem repeated RTX motifs fold into a characteristic parallel β-roll structure, as revealed by structural characterization of various RTX-containing proteins^3^,^41^. These parallel β-helices are highly regular super secondary structures stabilized by calcium ions^5^,^42^,^43^,^44^. The first six residues from each RTX motif (GGXGXD) form a turn involved in calcium binding while the last three residues (XUX) form a short β-strand within, with the conserved hydrophobic residues (U) collectively stabilizing the hydrophobic core of the β-helix (Fig. 3).

**Figure 6:**
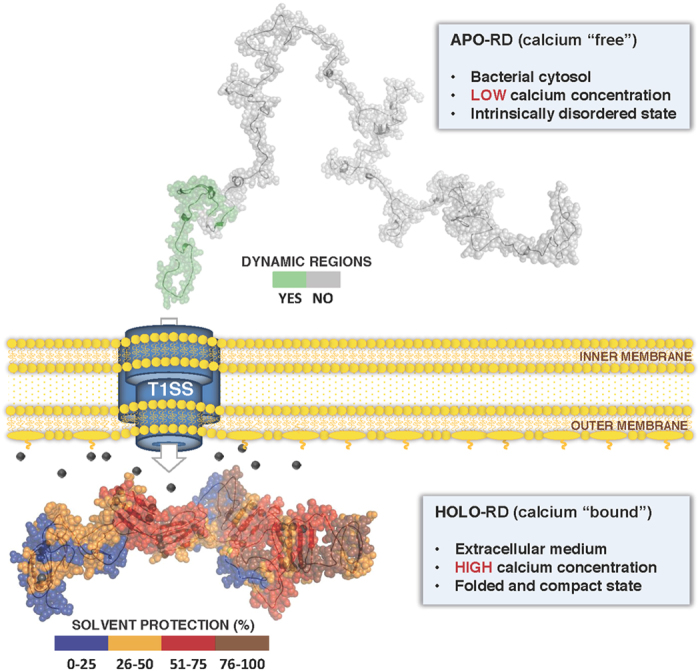
A structural model for the secretion of RTX proteins. Within bacteria, Apo-RD is an elongated and flexible disordered coil with a thin diameter appropriately sized for transport through the narrow channel of the bacterial secretion machinery. The secretion signal, the structure-containing F6 region and the unfolded Block V are the first regions to exit the T1SS into the calcium-rich environment of the bacterial extracellular milieu. Calcium binding to the RTX motifs induces folding of RD into a compact and stable structure larger than the T1SS conduit, thus ensuring an irreversible exit of the polypeptide. This disorder-to-order transition is essential for the secretion of RTX-containing toxins.

We used the Webserver Phyre2^45^ to model Holo-RD taking into consideration the calcium-induced solvent exposure reduction revealed by the HDX-MS experiments presented above. We obtained models for six protein regions (one per each RTX block, plus an extra one for the N-terminal flanking region of Block V). Using the Bunch program^46^, we refined as rigid bodies the position of those domains linked by chains of dummy residues so as to fit the experimental SAXS pattern of Holo-RD (see Supplementary Information for details). One hundred runs yielded as many models with similar global characteristics, but all differing in detail. A typical conformation is shown in Fig. 3B, together with the fit of the calculated scattering curve to the experimental data in panel C (χ = 1.08). In Fig. 6, we superimposed on this model the calcium-induced deuterium uptake reduction measured in the HDX-MS experiments.

From this representation, one can conclude that Holo-RD is likely to adopt an anisometric conformation in which distinct folded domains, corresponding to individual RTX blocks, are linked by structured regions, although part of them may exhibit some degree of restricted flexibility. Even the central region between Block III and IV that appears to be somewhat less compact and more accessible (green arrow in Fig. 3B), is far from being unfolded, in agreement with our HDX-MS results. Finally, we calculated the value of the sedimentation coefficient of our model using US-SOMO^47^ and obtained a value of 4.68 S, very close to the experimentally measured value of 4.5 ± 0.5 S^21^, providing an independent support to the validity of our model. While the protein is likely to explore a manifold of similar conformations through the restricted flexibility of essentially folded and compact linkers, no extra information could be obtained by describing Holo-RD using an ensemble of conformations as explained in the Materials and Methods section. Indeed, the unique model obtained by Bunch conveys the main features of Holo-RD conformations that are relevant for its biological function.

## Discussion

Here, we propose structural models (Fig. 6 and Supplementary Movie), based on combined SAXS, Raman spectroscopy and HDX-MS data, for both the unstructured, calcium-free Apo-state and the folded calcium-bound Holo-state of the RTX domain (RD) of the *B. pertussis* CyaA toxin. This data, in line with prior biophysical and thermodynamic studies, strongly supports the view that the disordered state of the RTX motifs within the bacteria may facilitate protein secretion through the T1SS while in the extracellular environment, calcium triggers folding of the toxin into its cytotoxic active form.

Our SAXS data indicate that in the absence of calcium, RD is well described using statistical polymer physics: Apo-RD adopts elongated and disordered conformations that can be modeled as a polymer chain made of *ca* 10 statistical elements, with an average length b of 80 Å and a diameter d of 13 Å. Yet, Apo-RD contains local, residual structural elements. This is evidenced by the averaged dimensions of the statistical elements of Apo-RD, that are significantly more collapsed than expected for a fully unfolded polypeptide (see Table S1). This data is in agreement with the ensemble of conformations generated by processing the SAXS data with the EOM program (see Fig. 2C). One of these conformations is used to illustrate and summarize the SAXS, Raman spectroscopy and HDX-MS findings (Fig. 6). Raman spectroscopy further confirms that Apo-RD is characterized by a high content of structural disorder and solvent-exposed aromatic residues, in agreement with previous data^21^, although it contains significant residual β-sheet structures at low temperature (20 °C). These β-sheet elements are largely unstable and essentially disappear above the melting temperature of Apo-RD (37 °C, see^22^). Our present SAXS experiments were performed at 15 °C and thus provide a typical picture of an intrinsically disordered protein containing residual structural elements. It is reasonable to propose that at the physiological temperature of 37 °C, Apo-RD may be more unfolded and flexible, as the residual secondary structures would all but vanish.

HDX-MS experiments allowed us to probe the local flexibility and structural dynamics of Apo-RD along the entire protein sequence (Fig. 5B). Deuterium uptake provides direct evidence that Apo-RD dynamics is characterized by two distinct regimes. The vast majority of the protein, from residues 1 to 600, exhibits a rapid and heterogeneous exchangeable amide pattern (Fig. 5B, 5E1 to 5E3). Such fast amide exchange, performed in a matter of seconds, is characteristic of fully solvent-exposed amide groups as typically found in intrinsically disordered polypeptides^39^,^40^. Only the last C-terminal 100 residues of Apo-RD present partly stable and solvent-protected secondary structures, as indicated by the dynamic nature of the HDX process (Fig. 5B, residues 600–700; Fig. 5E4 and 5E5). This C-terminal flanking region is emphasized by a green color on the Apo-RD conformation shown in Fig. 6.

Calcium binding induces dramatic structural changes of RD as revealed by the molecular shapes derived from the SAXS pattern: Holo-RD is an anisometric multi-domain protein with an elongated and curved shape comprising well-structured RTX Blocks with approximate dimensions of 40 Å in diameter. HDX-MS experiments revealed a massive masking effect, in particular in the RTX motifs, indicating that, in the presence of calcium, the RTX labile amide hydrogens are no longer exchangeable. This suggests that these amides are involved in the formation of stable and probably buried secondary structures. In agreement, Raman spectroscopy shows an increase in the amount of β-sheet content in the calcium-bound form of the protein at the expense of structural disorder and the transfer of aromatic residues into hydrophobic environments. These structural properties are clearly observed in the Phyre2/BUNCH model of Holo-RD (Fig. 3), which has been built from stable RTX Blocks, linked by folded and flexible flanking regions as indicated by the dynamic HDX regime of these inter-Block sequences in the presence of calcium (Fig. 5C). The Phyre2/BUNCH model is in excellent agreement with the GASBOR envelope.

The results presented herein provide structural insights into the adaptation of RTX motifs to the toxin secretion process. We have previously shown that RTX motifs are largely unstructured in the absence of calcium^21^,^22^,^24^ mainly because of the electrostatic repulsion between the negatively-charged residues of the RTX motifs and their overall low sequence hydrophobicity^25^. We proposed that these intrinsically disordered conformations may prevail in the low calcium environment of the bacterial cytosol ([Ca^2+^] ∼ 0.1–1 μM) and could thus favor the secretion of the polypeptide chain by the T1SS. This tripartite machinery, made of an inner-membrane ATP-binding cassette (ABC) transporter, a membrane fusion protein (MFP), and an outer membrane protein (OMP), forms a continuous channel across both the inner and outer membranes of the bacterial cell, and allows passage of the secreted RTX polypeptides directly from the cytosol to the extracellular medium. The RTX proteins have a C-terminal secretion signal (Figure S1) that is specifically recognized by the ABC transporter. It is generally assumed that the secretion proceeds vectorially from the C- to the N-terminus, with the C-terminal secretion signal reaching the extracellular side first.

Our present structural studies indicate that Apo-RD is an elongated and flexible disordered coil with a thin diameter (13 Å) most appropriately sized for transport through the narrow (20 to 30 Å^48^) channel of the bacterial secretion machinery. Conversely, in the calcium-rich external environment, Holo-RD adopts well-structured and stable conformations, as illustrated in Fig. 6. This data highlights the remarkable adaptation of RTX motifs to the structural constraints of the secretion process through the T1SS, and support our proposal that disorder-to-order transition experienced by RTX motifs upon calcium binding may favorably contribute to the secretion process. Indeed, assuming a directional C to N process for the secretion of RD, the C-terminal F6 and RTX Block V regions should be the first part of the polypeptide to exit the secretion channel into the calcium-rich extracellular environment. We previously showed that Block V, together with the F6 region, can autonomously fold in the presence of calcium into a compact and stable structure^23^,^25^. Once the Block V-F6 region is secreted and folded, it might then act as a nucleation site for the folding of the upstream RTX Blocks, as they progressively emerge from the secretion channel. Interestingly, HDX-MS experiments revealed a clear gradient of stability from the C-terminal to the N-terminal of the RD polypeptide chain.

Such a progressive folding process could improve the efficiency of the secretion mechanism of the RTX-proteins, as upon secretion and folding, the dimensions of each folded RTX Block (about 40-50 Å^23^,^25^) become larger than the diameter of the T1SS channel (about 20 Å or 30 Å backbone to backbone, as seen in the crystal structure of OMP protein^48^). This large change of RTX Block dimensions may prevent any backward movements of the polypeptide chain through the secretion channel, and thus biases its transport toward the extracellular side of the bacterium. The dimensions of the folded multi-domain Holo-RD (represented to scale with the T1SS in Fig. 6) therefore ensure the completion and irreversibility of the secretion of the polypeptide chain engaged in the T1SS channel. Once fully exported and folded, the RD domain may then serve as a folding nucleus for the upstream regions of the CyaA polypeptide, as they progressively exit the T1SS channel.

This co-secretional folding of CyaA at the mouth of the T1SS machinery may contribute to the confinement of individual toxin molecules at the bacterial surface, preventing their potential aggregation and thus favoring their folding as a monomeric active species^10^. The calcium-dependent disorder-to order transition of the RTX motifs thus appears as a simple, robust and very effective design to combine the structural constraints of protein secretion across a narrow secretory channel on the one hand, with the need for efficient folding of a large polypeptide chain in the absence of any chaperone in the extra-cellular environment on the other. This may explain the wide distribution and conservation of this type of structural motif in the large family of Repeat in ToXin (RTX) proteins that are found in more than 250 species of Gram-negative bacteria and cover a variety of biological functions^3^,^4^.

## Methods

Methods are described in Supplementary Information

## Additional Information

**How to cite this article**: O’Brien, D. P. *et al.* Structural models of intrinsically disordered and calcium-bound folded states of a protein adapted for secretion. *Sci. Rep.* **5**, 14223; doi: 10.1038/srep14223 (2015).

## Supplementary Material

Supplementary Information

Supplementary Movie
