# Supplementary material for: Structural models of intrinsically disordered and calcium-bound folded states of a protein adapted for secretion
Source: Sci Rep. 2015 Sep 16;5:14223. doi: 10.1038/srep14223 (PMC4642704; doi:10.1038/srep14223)
Supplement: Supplementary Information [file srep14223-s1.pdf]

Supplementary Information

Structural models of intrinsically disordered and calcium-bound folded states of a protein adapted for secretion

Darragh P. O'Brien, Belen Hernandez, Dominique Durand, Véronique Hourdel, Ana-Cristina Sotomayor-Pérez, Patrice Vachette, Mahmoud Ghomi, Julia Chamot-Rooke, Daniel Ladant, Sébastien Brier, Alexandre Chenal

MATERIALS AND METHODS

Buffers

Experiments were performed in 20 mM HEPES, 150 mM NaCl, pH 7.4 ± 0.1, unless stated otherwise.

Protein production and purification

RD protein was produced and purified as described previously [1-3]. RD is a 701 residue-long protein and corresponds to residues 1006 to 1706 of the CyaA toxin with an extra N-terminal methionine.

The primary sequence of RD is:

```
MLEHVQHIIGGAGNDSITGNAHDNFLAGGSGDDRLDGGAGNDTLVGGEGQ
NTVIGGAGDDVFLQDLGVWSNQLDGGAGVDTVKYNVHQPSEERLERMGDT
GIHADLQKGTVEKWPALNLFSVDHVKNIENLHGSRLNDRIAGDDQDNELW
GHDGNDTIRGRGGDDILRGGLGLDTLYGEDGNDIFLQDDETVSDDIDGGA
GLDTVDYSAMIHPGRIVAPHEYGFGIEADLSREWVRKASALGVDYYDNVR
NVENVIGTSMKDVLIGDAQANTLMGQGGDDTVRGGDGDDLLFGGDGNDML
YGDAGNDTLYGGLGDDTLEGGAGNDWFGQTQAREHDVLRGGDGVDTVDYS
QTGAHAGIAAGRIGLGILADLGAGRVDKLGEAGSSAYDTVSGIENVVGTE
LADRITGDAQANVLRGAGGADVLAGGEGDDVLLGGDGDDQLSGDAGRDRL
YGEAGDDWFFQDAANAGNLLDGGDGRDTVDFSGPGRGLDAGAKGVFLSLG
KGFASLMDEPETSNVLRNIENAVGSARDDVLIGDAGANVLNGLAGNDVLS
GGAGDDVLLGDEGSDLLSGDAGNDDLFGGQGDDTYLFGVGYGHDTIYESG
GGHDTIRINAGADQLWFARQGNDLEIRILGTDDALTVHDWYRDADHRVEI
IHAANQAVDQAGIEKLVEAMAQYPDPGAAAAAPPAARVPDTLMQSLAVNWR
```

The primary sequence of CyaA (with RD in bold letters):

MQQSHQAGYANAADRESGIPAAVLDGIKAVAKEKNATLMFRLVNPHSTSL
IAEGVATKGLGVHAKSSDWGLQAGYIPVNPNLSKLFGRAPEVIARADNDV
NSSLAHGHTAVDLTLSKERLDYLRQAGLVTGMADGVVASNHAGYEQFEFR
VKETSDGRYAVQYRRKGGDDFEAVKVIGNAAGIPLTADIDMFAIMPHLSN
FRDSARSSVTSGDSVTDYLARTRRAASEATGGLDRERIDLLWKIARAGAR
SAVGTEARRQFRYDGDMNIGVITDFELEVRNALNRRAHAVGAQDVVQHGT
EQNNPFPEADEKIFVVSATGESQMLTRGQLKEYIGQQRGEGYVFYENRAY
GVAGKSLFDDGLGAAPGVPSGRSKFSPDVLETVPASPGLRRPSLGAVERQ
DSGYDSLDGVGSRSFSLGEVSDMAAVEAAELEMTRQVLHAGARQDDAEPG
VSGASAHWGQRALQGAQAVAAAQRLVHAIALMTQFGRAGSTNTPQEAASL
SAAVFGLGEASSAVAETVSGFFRGSSRWAGGFGVAGGAMALGGGIAAAVG
AGMSLTDDAPAGQKAAAGAEIALQLTGGTVELASSIALALAAARGVTSGL
QVAGASAGAAAGALAAALSPMEIYGLVQQSHYADQLDKLAQESSAYGYEG
DALLAQLYRDKTAAEGAVAGVSAVLSTVGAAVSIAAAASVVGAPVAVVTS
LLTGALNGILRGVQQPIIEKLANDYARKIDELGGPQAYFEKNLQARHEQL
ANSDGLRKMLADLQAGWNASSVIGVQTTEISKSALELAAITGNADNLKSV
DVFVDRFVQGERVAGQPVVLDVAAGGIDIASRKGERPALTFITPLAAPGE
EQRRRTKTGKSEFTTFVEIVGKQDRWRIRDGAADTTIDLAKVVSQLVDAN
GVLKHSIKLDVIGGDGDDVVLANASRIHYDGGAGTNTVSYAALGRQDSIT
VSADGERFNVRKQLNNANVYREGVATQTTAYGKRTENVQYRHVELARVGQ
VVEVDT**LEHVQHIIGGAGNDSITGNAHDNFLAGGSGDDRLDGGAGNDTLV**
GGEGQNTVIGGAGDDVFLQDLGVWSNQLDGGAGVDTVKYNVHQPSEERLE
RMGDTGIHADLQKGTVEKWPALNLFSVDHVKNIENLHGSRLNDRIAGDDQ
DNELWGHDGNDTIRGRGGDDILRGGLGLDTLYGEDGNDIFLQDDETVSDD
IDGGAGLDTVDYSAMIHPGRIVAPHEYGFGIEADLSREWVRKASALGVDY
YDNVRNVENVIGTSMKDVLIGDAQANTLMGQGGDDTVRGGDGDDLLFGGD
GNDMLYGDAGNDTLYGGLGDDTLEGGAGNDWFGQTQAREHDVLRGGDGVD
TVDYSQTGAHAGIAAGRIGLGILADLGAGRVDKLGEAGSSAYDTVSGIEN
VVGTELADRITGDAQANVLRGAGGADVLAGGEGDDVLLGGDGDDQLSGDA
GRDRLYGEAGDDWFFQDAANAGNLLDGGDGRDTVDFSGPGRGLDAGAKGV
FLSLGKGFASLMDEPETSNVLRNIENAVGSARDDVLIGDAGANVLNGLAG
NDVLSGGAGDDVLLGDEGSDLLSGDAGNDDLFGGQGDDTYLFGVGYGHDT
IYESGGGHDTIRINAGADQLWFARQGNDLEIRILGTDDALTVHDWYRDAD

HRVEIIHAANQAVDQAGIEKLVEAMAQYPDPGAAAAAPPAARVPDTLMQS
LAVNWR

The Block A of RD [4] is a 15 amino-acid long sequence: `TDDALTVHDWYRDAD` located at position 1636-1650 in CyaA and 631-645 in RD and underlined in both sequences above.

Small Angle X-Ray Scattering

X-ray scattering data was collected at the SWING beamline of the SOLEIL Synchrotron (Gif-sur-Yvette, France) (see Table S1 for details). Measurements were performed using an online size-exclusion HPLC column (Agilent BioSEC-3) with the SAXS measuring cell (a 1.5 mm diameter quartz capillary contained in an evacuated vessel [5]. Apo-RD was prepared in 20 mM HEPES, NaCl 150 mM, 2 mM DTT, pH 7.5; Holo-RD was prepared in the same buffer supplemented with Ca^{2+} (4 mM final concentration). Briefly, 50 µL of an 8 mg/mL sample solution was loaded onto the equilibrated column. Scattering of the elution buffer before void volume was recorded and used as buffer scattering for subtraction from all protein patterns. Successive frames of 1 s were recorded separated by a 0.5 s interval. The elution flow of 0.2 mL/min ensured that no protein was irradiated for more than 0.4 s.

Primary data reduction was performed using Foxtrot, the SWING in-house software. This yielded azimuthally averaged scattering intensities I(q) determined on the absolute scale using water scattering, where q is the momentum transfer ($q = 4\pi \sin\theta/\lambda$, where 2θ is the scattering angle and λ the wavelength of the X-rays). Data was subsequently processed using the program package PRIMUS [6]. The forward scattering I(0) and the radius of gyration (R_g) were evaluated using the Guinier approximation [7]. Frames over the elution peak were analyzed individually before averaging the appropriate subset that yielded identical I(q)/c profiles. The corresponding concentration was determined using the UV absorbance detector from the HPLC system and the values of the protein absorbance at 280 nm ε_{280}= 73,340 $M^{-1}cm^{-1}$. The distance distribution function P(r) was determined using the indirect Fourier transform method as implemented in the program GNOM [8]. The molecular mass was estimated from the value of the forward scattering. An alternative estimate of the molecular mass was obtained for Holo-RD using the SAXSMoW program available at the URL http://www.if.sc.usp.br/~saxs/ that is based on the determination of the Porod volume [9], which is independent of the sample concentration.

While scattering curves of Apo-RD were essentially identical through the elution peak, the successive frames through the main peak of the Holo protein exhibited continuous changes

with gradual reduction of the radius of gyration, D_{max} and molecular mass values. The latter decreased from 1.4 M_m down to about M_m where M_m is the calculated mass of the monomer, suggesting that the eluted solution contained both monomers and dimers of the protein in equilibrium. Using the program GASBORMX [10] we globally fitted an ensemble of slices through the elution peak (each slice was the average of five successive frames to improve statistics) as an equilibrium between a monomer and a symmetric dimer. The volume fraction of dimer decreases from 30% in the earliest slice to 0% for the last slice on the falling edge of the peak (protein concentration c=0.38 g/L). We therefore considered that the latter curve was that of the monomer of Holo-RD and used it for further analysis. This was later confirmed by recording the scattering pattern of a dilute (c=0.15 g/L) solution of Holo-RD, top of the elution peak from a SE-HPLC column loaded with a more dilute solution. The curve was identical to that obtained at the end of the elution peak.

Modelling Apo-RD using EOM

We undertook Apo-RD modeling in terms of ensembles of conformations using the package EOM (Ensemble Optimization Method) [11,12]. This is a well-suited representation for a highly flexible, essentially unstructured protein such as Apo-RD. The program Ranch within EOM creates a large (10,000) pool of random conformations using a chain of dummy residues describing the protein. The program offers a choice between three chain types to be generated corresponding to the use of three Cα angle distributions: random coil, native and compact. On average, random models will be more extended than native-like, while the "compact" option will force the reconstructed linkers to be rather compact. Dummy residues are subsequently substituted by complete residues using the programs PD2 [13] and SCWRL4 [14] before calculating all scattering patterns using Crysol [15]. The routine Gajoe within EOM tries to fit the experimental scattering curve by the average of the calculated scattering patterns of an ensemble of conformations using a genetic algorithm protocol. The program is run many times and yields equally good fits with different ensembles. The resulting conformations in any ensemble should therefore be considered as illustrations of the polypeptide chain main features, rather than actual conformations adopted by the protein.

Holo-RD modeling using Phyre2 and Bunch

The Web server Phyre2 (http://www.sbg.bio.ic.ac.uk/~phyre2/html/page.cgi?id=index) [16] was used to model the structure of RD regions for which HDX-MS experiments revealed a masking effect induced by calcium binding, mainly in Blocks II to V. Indeed, the β-helix

model proposed by Phyre2 for these regions can be considered to be reasonably robust [17]. Furthermore, several runs of Phyre2 were performed and only the structured regions common to all models were retained. Block I was also modeled by Phyre2 although it did not exhibit any masking effect in the presence of calcium. Finally, we considered that Phyre2 modeling was reliable in the following regions: 1-70 (within Block I), 137-185 (within Block II), 258-324 (in Block III), 391-459 (Block IV), 478-525 (N-ter flanking region to Block V) and 530-604 (Block V). Calcium atoms were positioned close to the consensus binding sites. We then ran the Bunch program to obtain a model of the protein that fits the experimental scattering curve of Holo-RD using rigid body movements of the above six structured regions joined by linkers described as chains of dummy residues [18]. In a final step, we substituted the dummy residues with all atom descriptions using the programs PD2 and SCWRL4.

Holo-RD modeling using EOM

While the unique model obtained by Bunch conveys the main features of Holo-RD conformation, the protein is likely to explore a manifold of similar conformations through the restricted flexibility of the essentially folded and compact linkers, suggesting that an ensemble of conformations might be a more appropriate description. We therefore tried to fit the scattering pattern of Holo-RD using EOM in the "compact mode". However, all our attempts failed to fit the data. We believe that this is due to the parameters of the program that, even in the so-called "compact" mode, builds fairly extended linker conformations. Therefore, if two domains are close to one another, the intervening linker often adopts an implausible "hairpin-like" conformation instead of adopting a locally collapsed arrangement of residues. Since most domains are fairly close to one another in the Bunch models of Holo-RD, the program fails in finding the very few "compact" conformations that, in any event, present unlikely conformations of linkers. This failure does not point towards a limitation of the EOM program that had not been designed for modelling compact proteins, but rather underlines compactness as a key feature of the configuration adopted by Holo-RD, likely accompanied by some degree of restricted flexibility of significantly collapsed linkers.

Raman spectroscopy

Raman spectra were recorded by exciting RD aqueous solutions (5 mg/mL) in suprasil quartz cells (5 mm path length) with the 488 nm line of an Ar^+ laser (Spectra Physics). The exciting power at the sample was *ca* 200 mW. Scattered light at right angle was analyzed on a Jobin-Yvon T64000 in a single spectrograph configuration with a 1200 grooves/mm holographic

grating and a holographic notch filter. A liquid nitrogen cooled CCD detection system (Spectrum One, Jobin-Yvon) was used to collect Raman data. The effective spectral slit width was set to *ca* 5 cm^{-1}. Each spectrum corresponds to a total acquisition time of 1200 s. The HEPES buffer was exchanged by buffer exchange on G25SF columns for a Tris buffer: 20 mM Tris, 150 mM NaCl, pH 7.4. Stokes Raman spectra were analyzed as a function of temperature, in the 1750-400 cm^{-1} region (Figure S2). Spectra from Apo and Holo states were normalized to the 1450 cm^{-1} band (aliphatic CH_2/CH_3 bending motions). Curve fitting was performed on Amide I, Amide III and regions containing characteristic contributions from aromatic side chains (*i.e.*, 1100-950 and 925-725 cm^{-1}). Gaussian+Lorentzian functions, with Lorentzian contribution kept ≥ 50% were used. Post processing of Raman spectra was done with GRAMS/32 package (Galactic Industries).

Analysis of secondary structure

The profile analysis of amide I (1700-1600 cm^{-1}) and amide III (~1320-1220 cm^{-1}) regions allow confident estimation of secondary structure populations (Figures 4 and S4). It is worth noting that the spectral ranges corresponding to canonical structural elements in the amide I region (*i.e.*, α at *ca* 1650 cm^{-1}, β at *ca* 1670 cm^{-1}) may also contain contributions from different types of β-turns or random conformers. The simultaneous decomposition of amide III band provides additional information leading to a more unambiguous quantification. Despite the fact that amide III may contain minor contributions from amino acids side chains as well as from CCH bending modes (mainly involving C_α), it is possible to distinguish them as amide III components that completely vanish upon deuteration.

Amino acid side chains

Changes in the environment of tyrosine side chains (Apo and Holo states) are deduced from the evolution of the integrated intensity ratio of the 850 and 830 cm^{-1} bands [19,20], obtained from curve fitting of the 925-725 cm^{-1} spectral region (Figure S3). The calculated ratios reflect the mean effect on the 16 Tyr residues in RD. The I_{850}/I_{830} ratio is strongly dependent of the hydrophobic/hydrophilic balance of interactions involving the tyrosine phenol ring. Solvent-exposed side chains typically show I_{850}/I_{830} ratios ranging between 0.9 and 1.4, while values lower 0.9 (in the 0.9-0.3 interval) are associated to side chains mostly involved in hydrophobic contacts. Band intensities arising from cycle vibrations in aromatic side chains are sensitive to the hydrophobic/polar character of the environment. For instance, Phe side chain gives a stronger 1003 cm^{-1} in weakly polar environments [21]. A similar behavior is found

for the 877 and 1360 Trp bands [20,21]. An opposite behavior is found for the Trp indole breathing vibration, appearing around 760 cm^{-1}, which increases with decreasing hydrophobicity [21]. Additional information can be extracted from the position of the ~ 880 cm^{-1} band, sensitive to H bond interactions involving the indolic N-H [21,22]. It is generally accepted that this band shift towards lower wavenumbers as the H bond strength increases. The band appears at 877 cm^{-1} in the case of solvent exposed residues or in the presence of NH… π interactions. Extremely hydrophobic environments can induce shifts up to 889 cm^{-1} [23].

HDX-MS

Sample preparation for HDX analyses

Purified RD protein (27 µM) was dissolved in 20 mM HEPES, 150 mM NaCl, pH 7.4 containing either 4 mM EGTA (Apo-RD) or 4 mM $CaCl_2$ (Holo-RD). Each RTX motif of RD binds to calcium at low millimolar affinities [3]. The use of 4 mM $CaCl_2$ ensures that the complex is fully formed and maintained during labeling. Prior to addition of the deuterated buffer, all solutions were equilibrated for 1 h at room temperature.

For intact protein analysis, continuous labeling was performed at room temperature for t = 0.16, 0.5, 1, 5, 10, 30, 60, 120 and 240 min, using two separate buffers of 99.9 % D_2O in 20 mM HEPES, 150 mM NaCl, pD 7.4, in either 4 mM EDTA or $CaCl_2$, respectively. An 80% excess of deuterium was selected such that the kinetics favored unidirectional exchange. Aliquots of 40 pmol of protein were withdrawn at each experimental time point and quenched upon mixing with ice-cold 0.5% formic acid solution to achieve a final pH of 2.5. Quenched samples were immediately snap-frozen in liquid nitrogen and stored at −80 °C for approximately 12 h. Undeuterated controls were treated using an identical procedure.

For local HDX analysis, samples were prepared in the presence of 4 mM EDTA or $CaCl_2$, labeled for t = 0.16, 0.5, 1, 2, 5, 8, 10, 15, 30, 60, 120, 240 and 480 min, quenched and stored as above. Here, 15 pmol of protein was used per analysis. Fully deuterated samples of RD were achieved after overnight incubation at 50°C in the presence of 1.3 M guanidinium chloride (final D_2O content of 80%) [3]. Triplicate analyses were performed for each time point and condition for all HDX-MS analyses.

Data acquisition and processing

Prior to mass spectrometric analysis, samples were rapidly thawed and immediately injected into a cooled nanoACQUITY UPLC HDX system (Waters) maintained at 0°C. For intact mass measurement, samples were loaded onto a Vanguard C4 pre-column (BEH, 1.7 µm, 2.1 x 5 mm, Waters) and desalted for 2 min at 100 µL/min with 95% solvent A (0.15% formic

acid, pH 2.5) and 5% solvent B (100% acetonitrile, 0.15% formic acid, pH 2.5). Proteins were eluted from the trap column over a 2 min gradient of 5-90% solvent B at 100 µL/min. For peptide analysis, samples were on-line digested using an in-house prepared cartridge of immobilized pepsin beads (Thermo Scientific, Rockford, IL) for 2 min at 70 µL/min and 20°C. Peptic peptides were rapidly trapped and desalted on a Vanguard C18 pre-column (BEH 1.7 µm, 2.1 x 5 mm, Waters) as above, and separated using an analytical ACQUITY UPLC™ BEH C18 column (BEH, 1.7 µm, 1 x 100 mm, Waters). Peptides were separated over a 10 min gradient of 5-40% solvent B at 38 µL/min. After each run, the pepsin column was manually cleaned with two washes of 0.8% formic acid, 5% acetonitrile, 1.5 M guanidinium chloride, pH 2.5. Blank injections were performed between each sample to confirm the absence of carry-over.

The LC flow was directed to a Synapt™ G2-Si HDMS™ mass spectrometer (Waters) equipped with a standard electrospray ionization (ESI) source. Mass accuracy was ensured by continuously infusing a Glu-1-Fibrinogen solution (100 fmol/µL in 50% acetonitrile) through the reference probe of the ESI source. Mass spectra were acquired in positive-ion and resolution mode over the *m/z* range of 50–1800. Peptides were identified in undeuterated samples using a data-independent acquisition scheme (MS^E), whereby mass information is collected at both low and high collisional energies for collisional induced dissociation. Peptide identifications were made by database searching in ProteinLynx Global Server 3.0 (Waters) and each fragmentation spectrum was manually inspected for assignment validation.

DynamX 3.0 HDX software (Waters) was used to extract the centroided masses of all peptides selected for local HDX-analyses; only one charge state was considered per peptide. No adjustment was made for back-exchange and the results are reported as relative deuterium exchange levels expressed in either mass unit or fractional exchange. Fractional exchange data was calculated by dividing the experimentally measured uptake by the theoretically maximum number of exchangeable backbone amide hydrogens that could be replaced into each peptide This number corresponds to the number of amino acid residues present in the peptide minus the number of proline residues and minus one for the N-terminus that back-exchanges too rapidly to be measured by MS [24]. HDX-MS data of overlapping peptides was used to refine the location of the deuterium content only if the back-exchange of the fully-deuterated overlapping peptides was similar (mean variation of 5.3%). HDX-data were visualized using an in-house designed tool created in R Studio (Véronique Hourdel *et al.*, *unpublished data*).

Supplementary Discussion

Heterogeneous HDX pattern of the RTX Blocks

Apo-RD exhibits a heterogeneous HDX pattern within the RTX Blocks, most significantly in Blocks II to V. Indeed, the accessibility of the amide groups of the RTX motifs from the C-terminal part of each RTX Block is low compared to that of the RTX motifs located at the N-terminus of the RTX Blocks (Figure 5B). The lower deuterium uptake in the C-terminal RTX motifs of the Blocks is probably due to local collapses in these regions. Interestingly, this heterogeneous HDX pattern appears to be correlated with the relative homology observed between the particular RTX motifs and the canonical RTX sequence, GGXGXDXUX, as the C-terminal motifs of each Block are globally more canonical than the N-terminal ones (see alignment in Figure S7). Hence, these results suggest that the canonical RTX motifs may exhibit more stable local structures (*e.g.*, with lower amide hydrogen exchanges) than the less conserved RTX sequences. Accordingly, upon calcium binding, the relative masking effect on RTX motifs is highest at the N-terminal RTX motifs within all RTX Blocks. This is highlighted in Figures 5D and S7. We propose that the conserved RTX motifs (labeled HL in pink boxes, Figure S7) of each RTX Block act as folding nuclei upon calcium binding and that the folding is propagated to the less-conserved N-terminal RTX motifs. On a larger scale, we observe a gradient of masking effect from Block V to Block I, or alternatively, two groups of RTX Blocks, Block V to IV and Block III to I, separated by a calcium insensitive flanking region (Figure S7).

The Block A: a critical structural element of RD

The HDX-MS data provide new information on the C-terminal flanking region F6 and on Block A, a stretch of 15 amino acids from the F6 region, encompassing residues 1636-1650 of CyaA (residues 631-645 in RD). Functional and structural complementation assays showed that the F6 region of RTX Block V is required for the calcium-induced folding of Block V and to restore both hemolytic and translocation activities of truncated CyaA toxins [1,4,25]. The HDX-MS data showing that the F6 region is intrinsically structured in Apo-RD suggests that it may act as a nucleation site for the calcium-induced folding of RTX Block V.

We have previously shown that Block A does not bind calcium by itself, but is crucial for the folding of full-length CyaA and of any derived RTX polypeptide [1,25,26]. The requirement of Block A for folding is poorly understood. It is noteworthy that Block A exhibits a massive

masking effect upon calcium addition. This indicates that Block A, which is rather accessible in the Apo-state, becomes hidden within Holo-RD. Hence, Block A performs a critical structural function, probably as a folding interface between neighboring regions. The molecular mechanism, by which Block A contributes to assemble together its neighboring regions, still remains to be elucidated.

Supplementary Figures

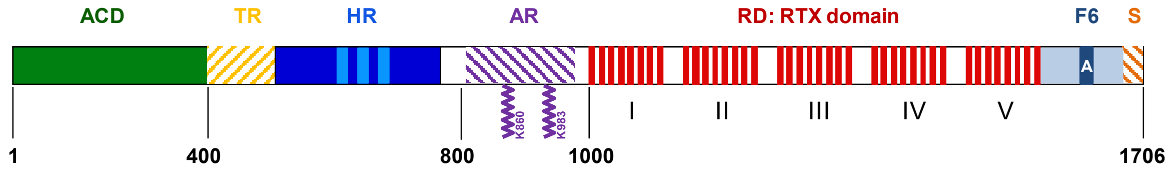

Figure S1. Schematic representation of the primary structure and regions of CyaA. CyaA is organized into several functionally-distinct regions. ACD: adenylate cyclase, catalytic domain; TR: translocation domain; HR: hydrophobic domain; AR: acylation domain with the post-translationally acylated Lys residues 860 and 983 displayed; RD: cell-receptor binding domain, RTX domain made of five (I–V) blocks of 8-10 RTX motifs; F6: Flanking region of Block V containing Block A (A); S: secretion signal.

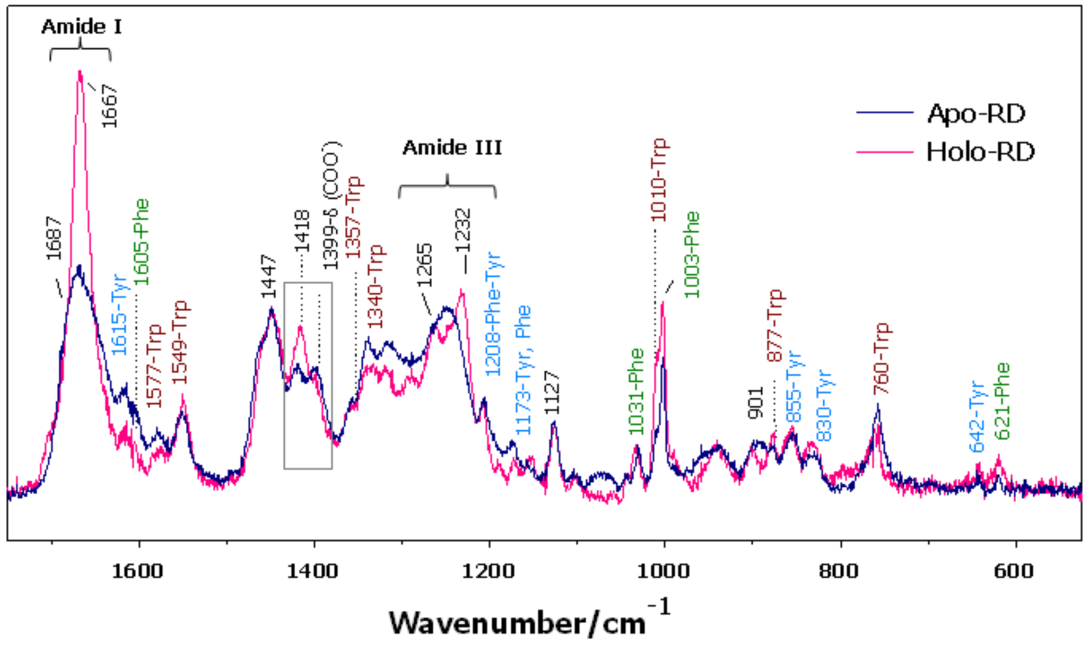

Figure S2. Full Raman spectra of RD. Stokes Raman spectra obtained at T=20°C of Apo-RD and Holo-RD solutions at 5g/L. Amide I and Amide III regions, as well as characteristic bands from aromatic amino acid side chains are indicated.

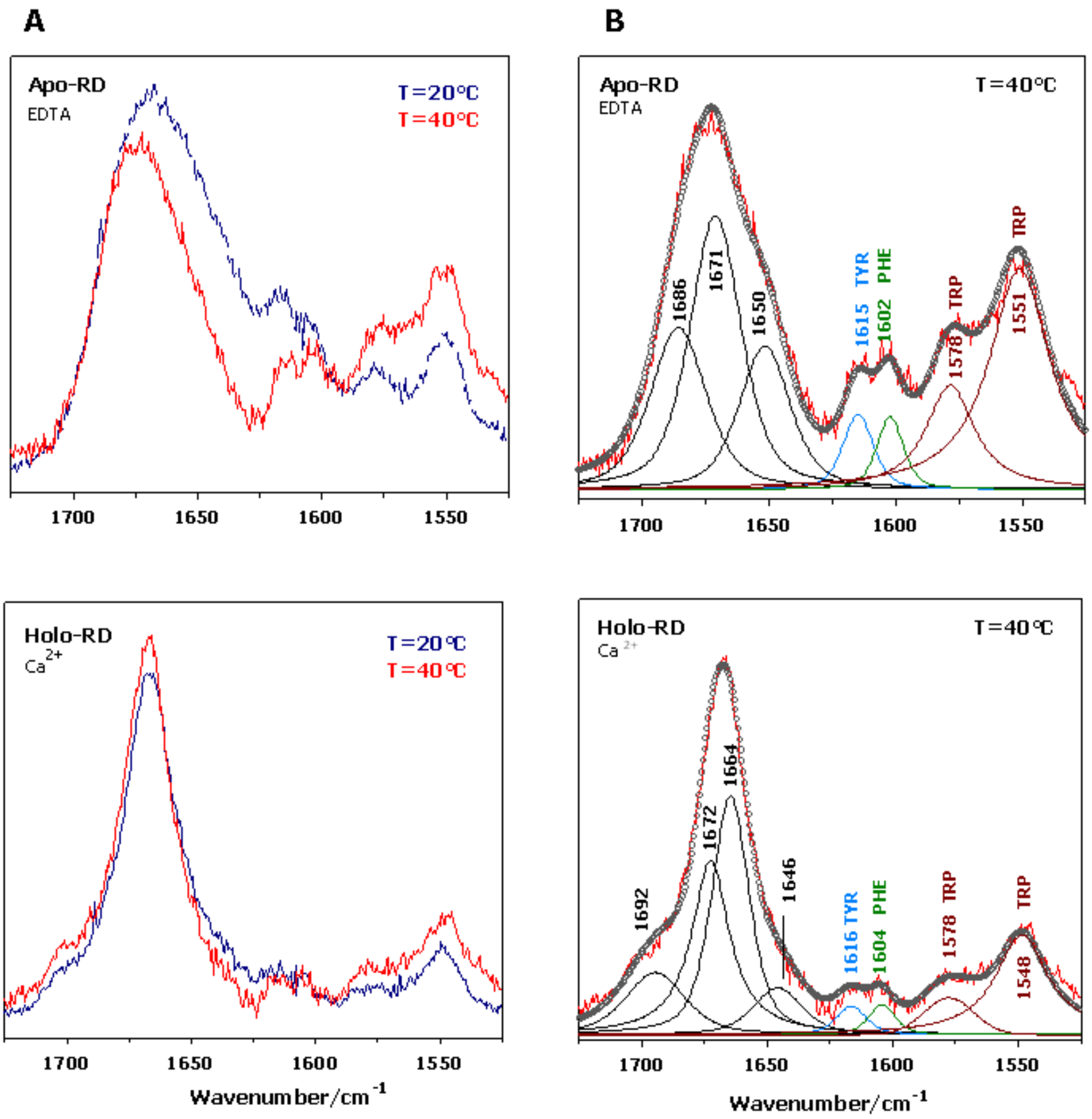

Figure S3. Effect of temperature on the secondary structure content of Apo-RD and Holo-RD. Panel A: Comparison of the amide I Raman profiles of RD at T=20 °C and T=40 °C (top: Apo form; bottom: Holo form). Panel B: Band decomposition of the amide I region from RD Raman spectra obtained at T=40 °C. See Table S2 and S3 for details.

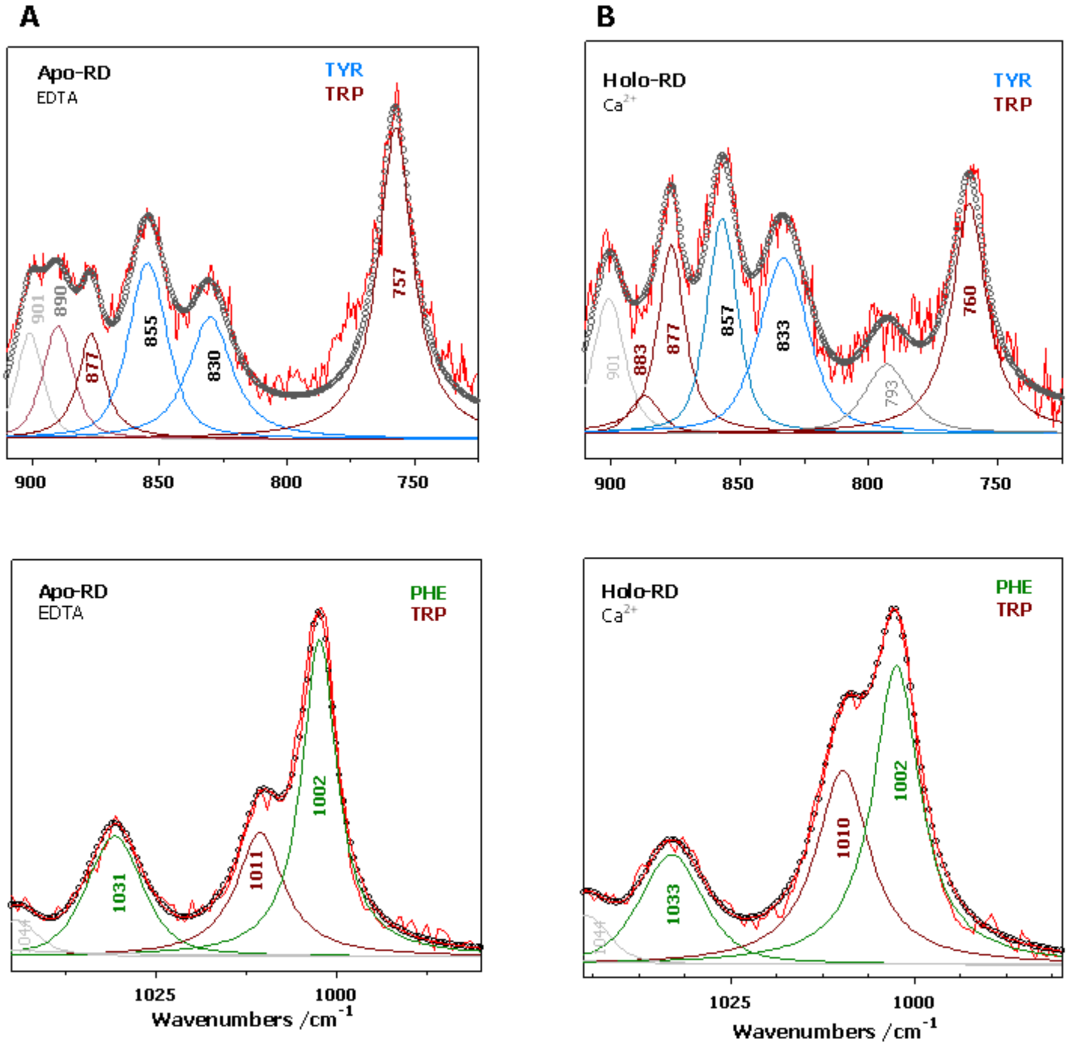

Figure S4. Band decomposition of the 925-700 cm^{-1} and and 1050-975 cm^{-1} Raman spectral regions of Apo-RD and Holo-RD containing relevant information on Tyr, Trp and Phe environments. See Table S4 for details.

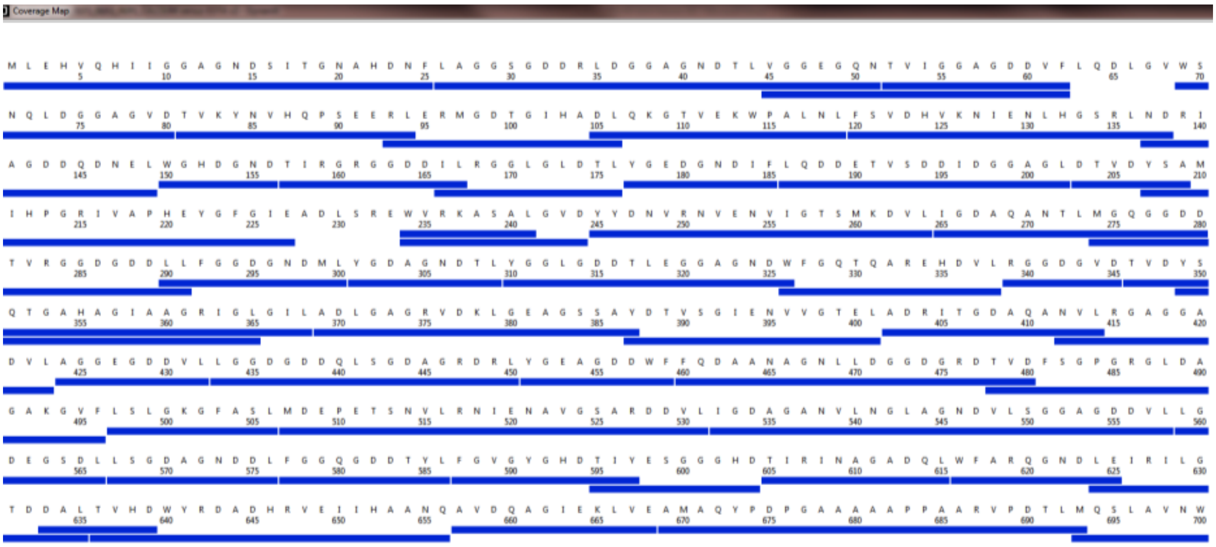

Figure S5. Peptide map of RD. HDX analysis was performed at the peptide level to improve the spatial resolution of the approach. A sequence coverage map of RD was determined after 2 min pepsin digestion. Each blue bar represents a single RD peptide. Linear sequence coverage of 98.1% was achieved.

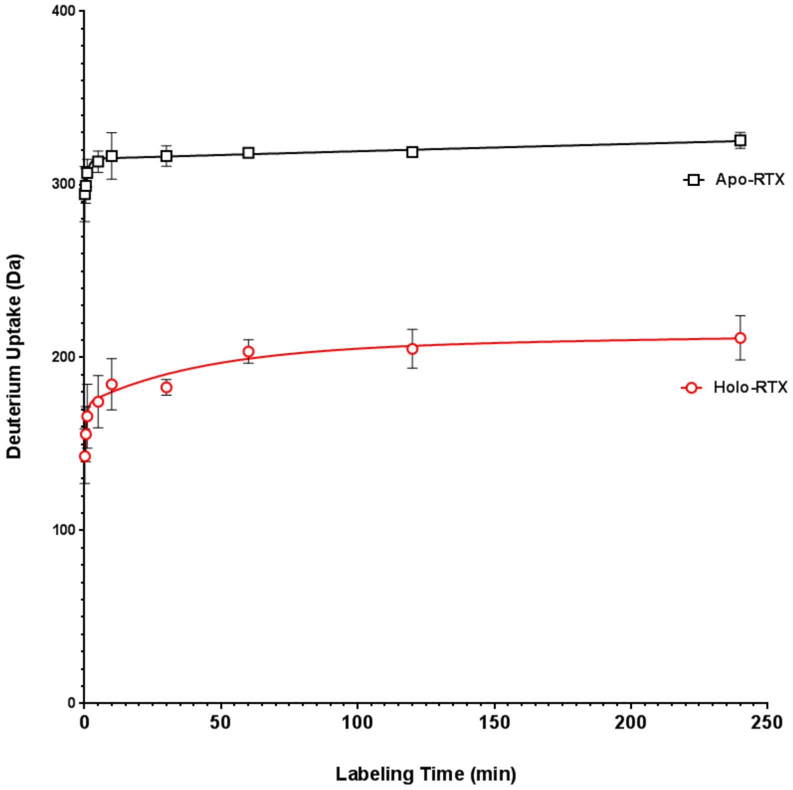

Figure S6. Global HDX-MS analysis of RD. HDX analysis was performed at the intact protein level to provide initial insights into conformation and in-solution dynamics of the protein. An increase in deuteration of RD was observed in the Apo-state, indicative of dynamic events and thus, residual secondary structural elements. Upon calcium addition, a substantial reduction in deuteration was observed, suggesting a large conformational change in the protein. Approximatively 40% of the exchangeable amide hydrogens of Holo-RD remained protected after 4 h incubation. All experiments were performed in triplicate.

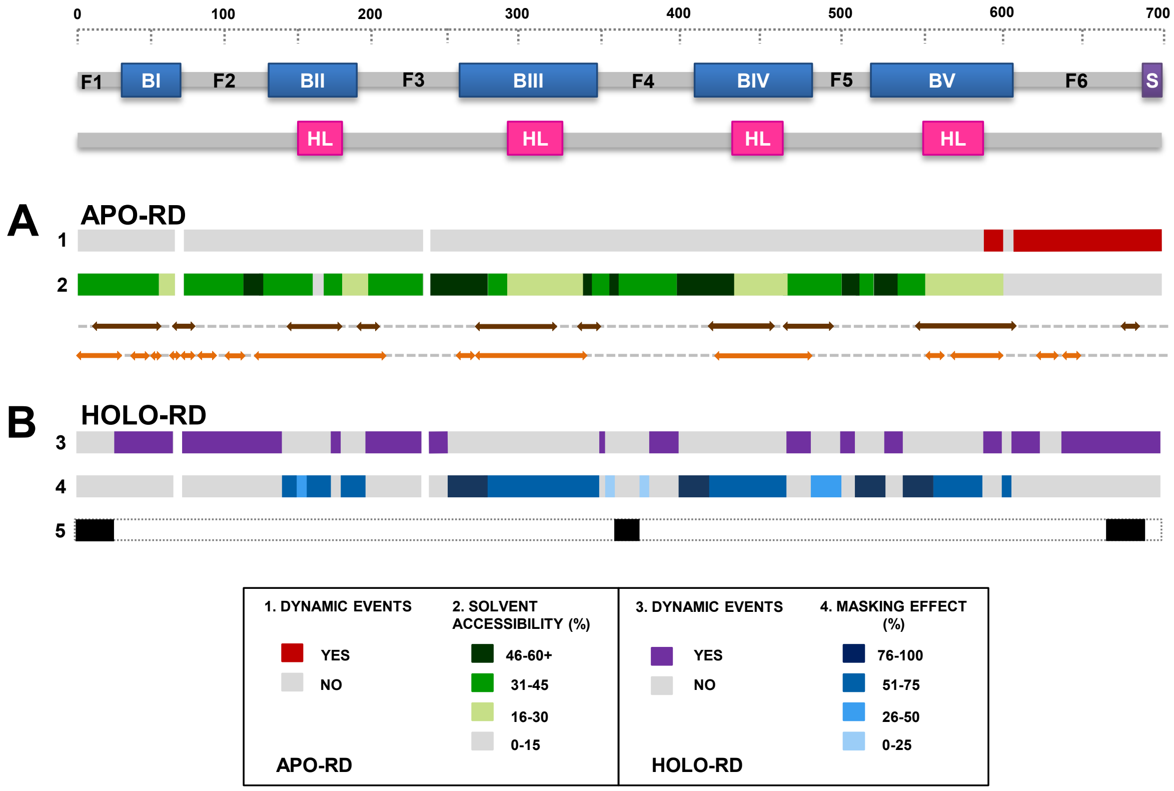

Figure S7. Summary of HDX analyses obtained for Apo- and Holo-RD. (A) For Apo-RD, dynamic events are highlighted in red (1), while the solvent accessibility is colored green (2). (B) For Holo-RD, dynamic events are colored in purple (3), masking effects in blue (4). Masking effect is expressed as the average protection factor calculated over all of the timepoints and is only determined for those regions which had no dynamic events in either condition. Regions unchanged in the absence and in the presence of calcium are colored in black (5). Regions of predicted structural disorder obtained from GlobProt2 (brown) and FoldIndex (orange) algorithms are aligned in-between the Apo and Holo data. For ease of comparison between datasets, the schematic representation of the RD protein, as described in Figure HDX 1.A, is displayed at the top of the figure. The motif HEMOLYSIN_CALCIUM (PS00330) corresponding to the RTX motif D-x-[LI]-x(4)-G-x-D-x-[LI]-x-G-G-x(3)-D is shown as pink boxes “HL”. A more detailed break-down of the data is provided in the color legend at the bottom of the figure.

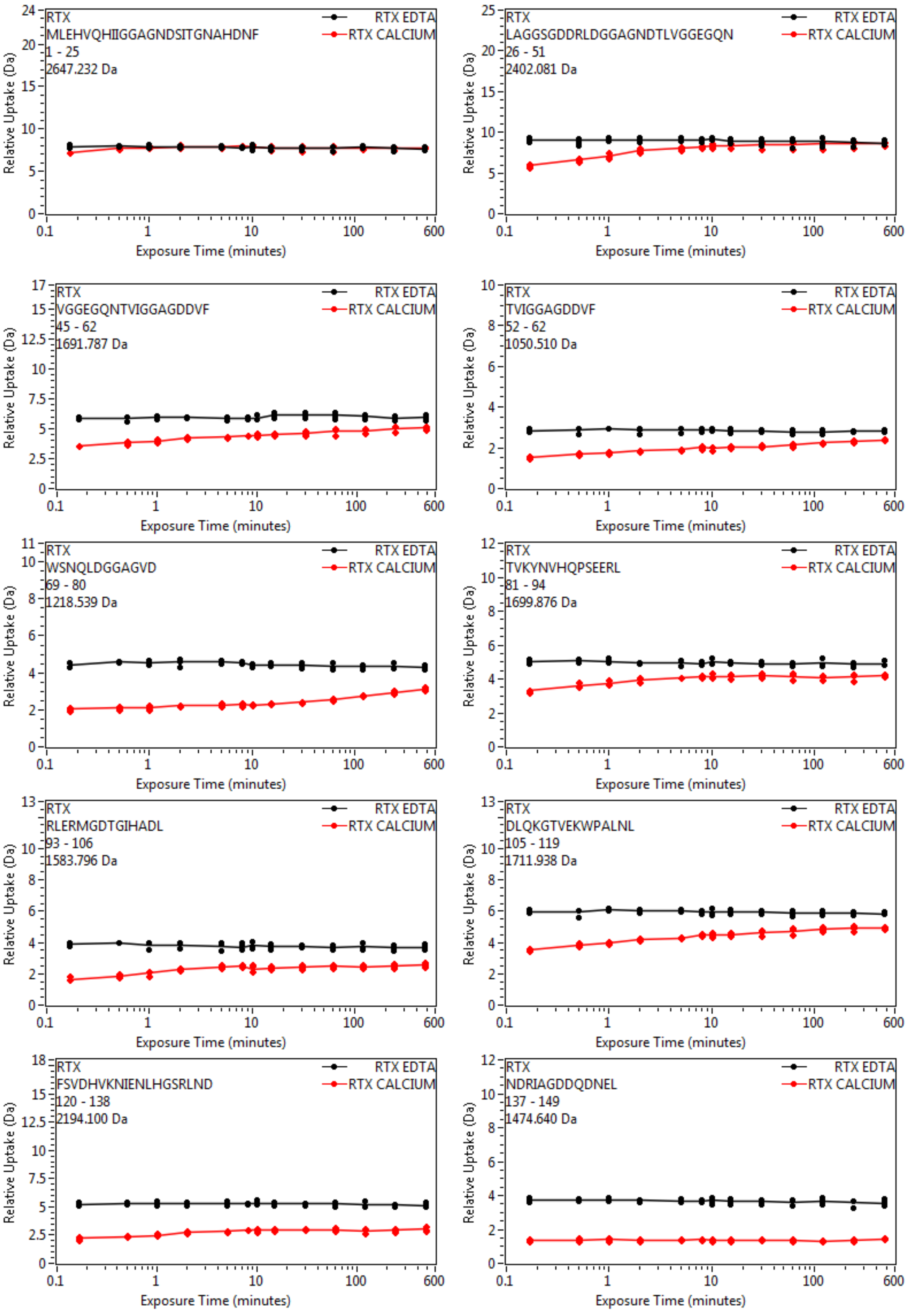

Figure S8. HDX-MS results for individual RD peptides across all conditions. For each peptide, the Apo form of the protein (RTX EDTA) is colored black, while the Holo form (RTX CALCIUM) is colored red. All experiments were performed in triplicate.

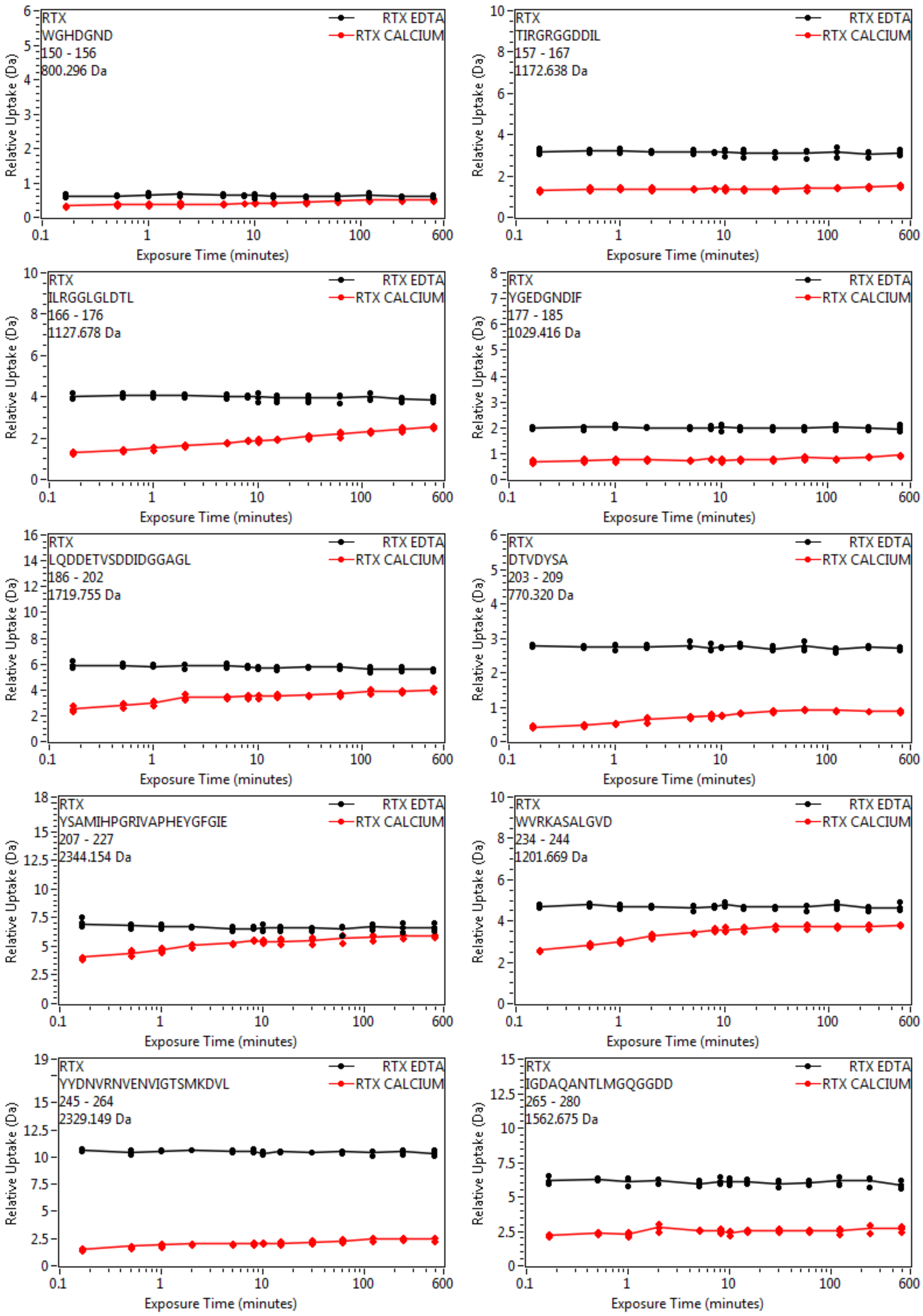

S8. (continued). HDX results for individual RD peptides across all conditions.

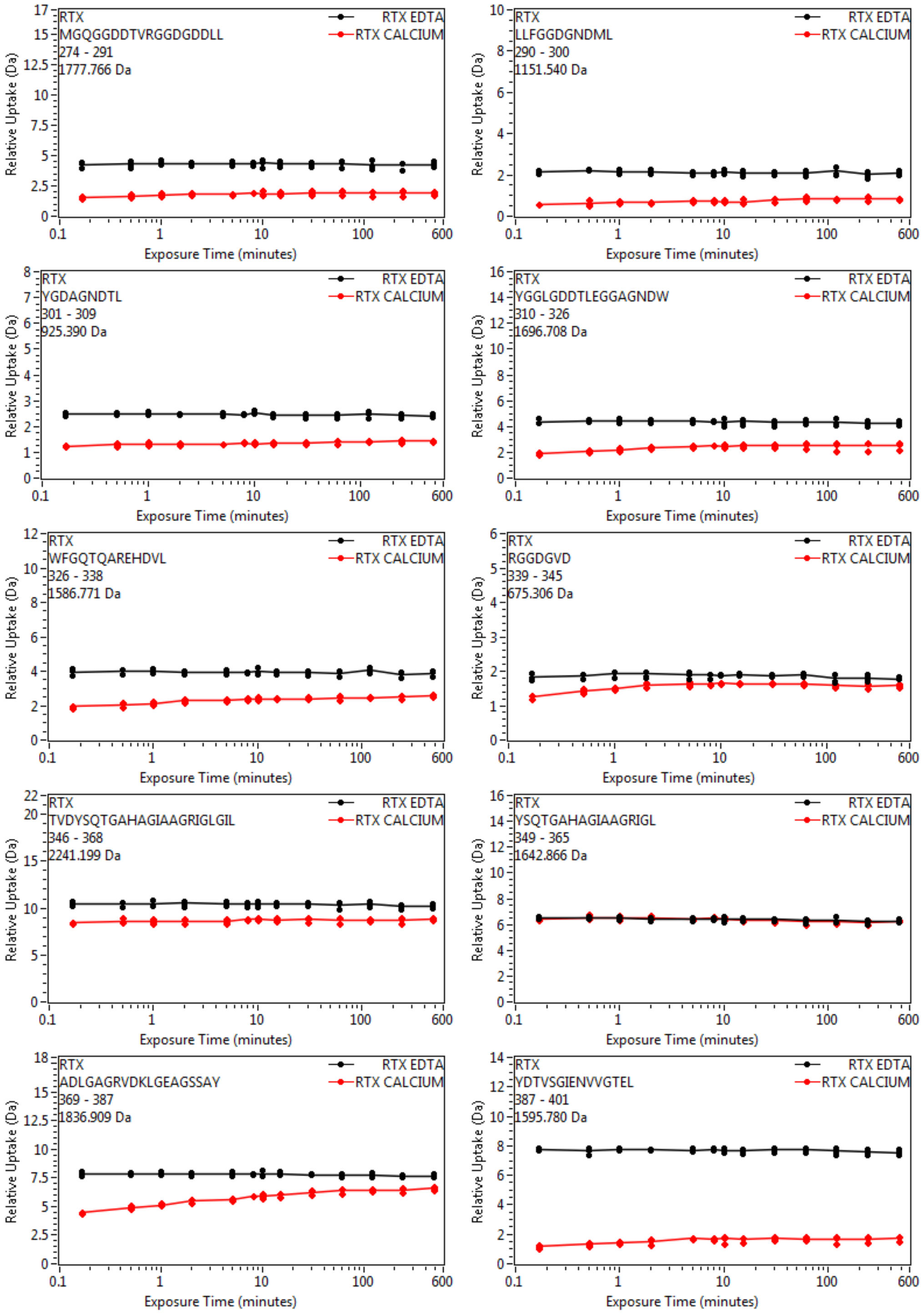

S8. (continued). HDX results for individual RD peptides across all conditions.

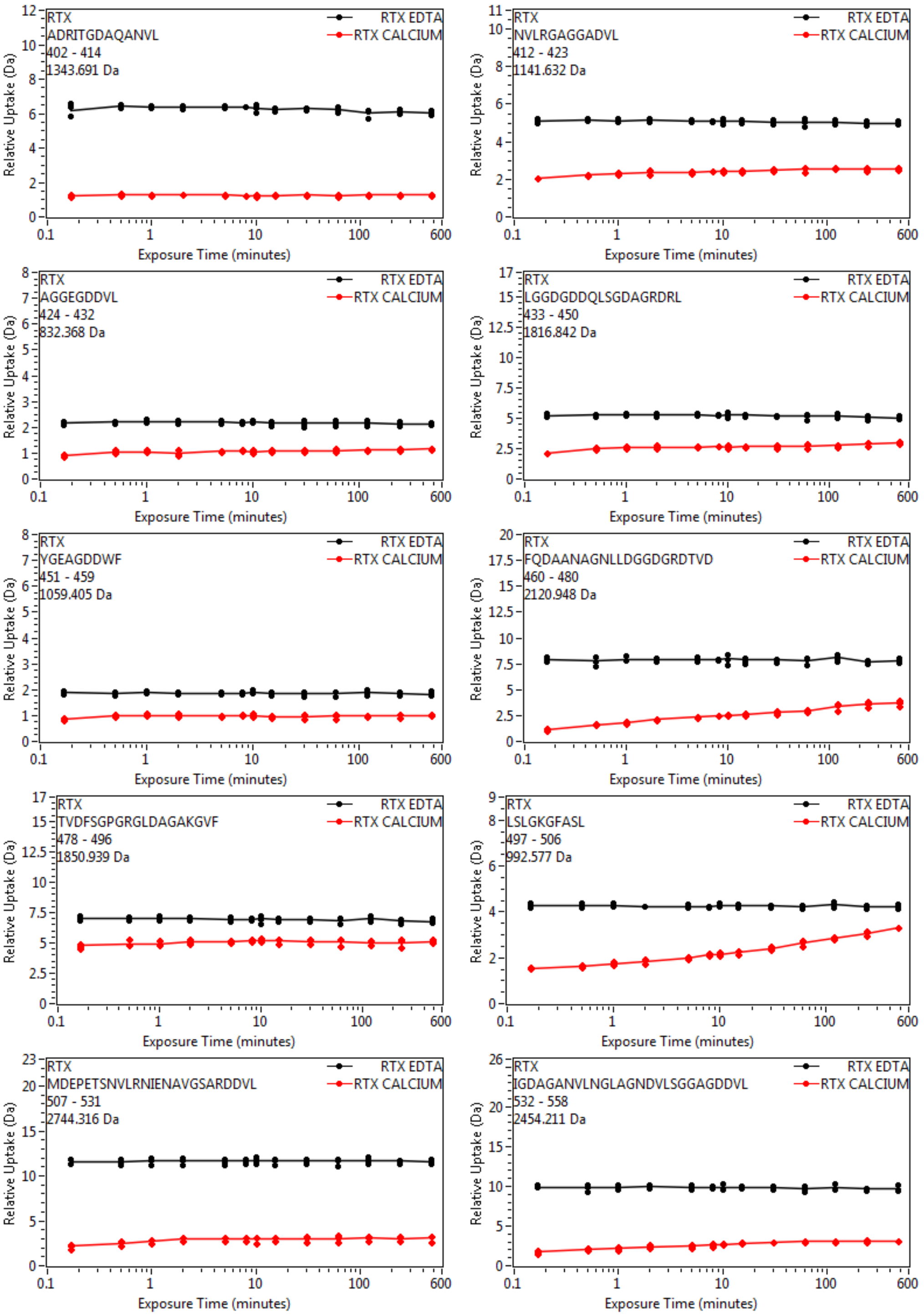

S8. (continued). HDX results for individual RD peptides across all conditions.

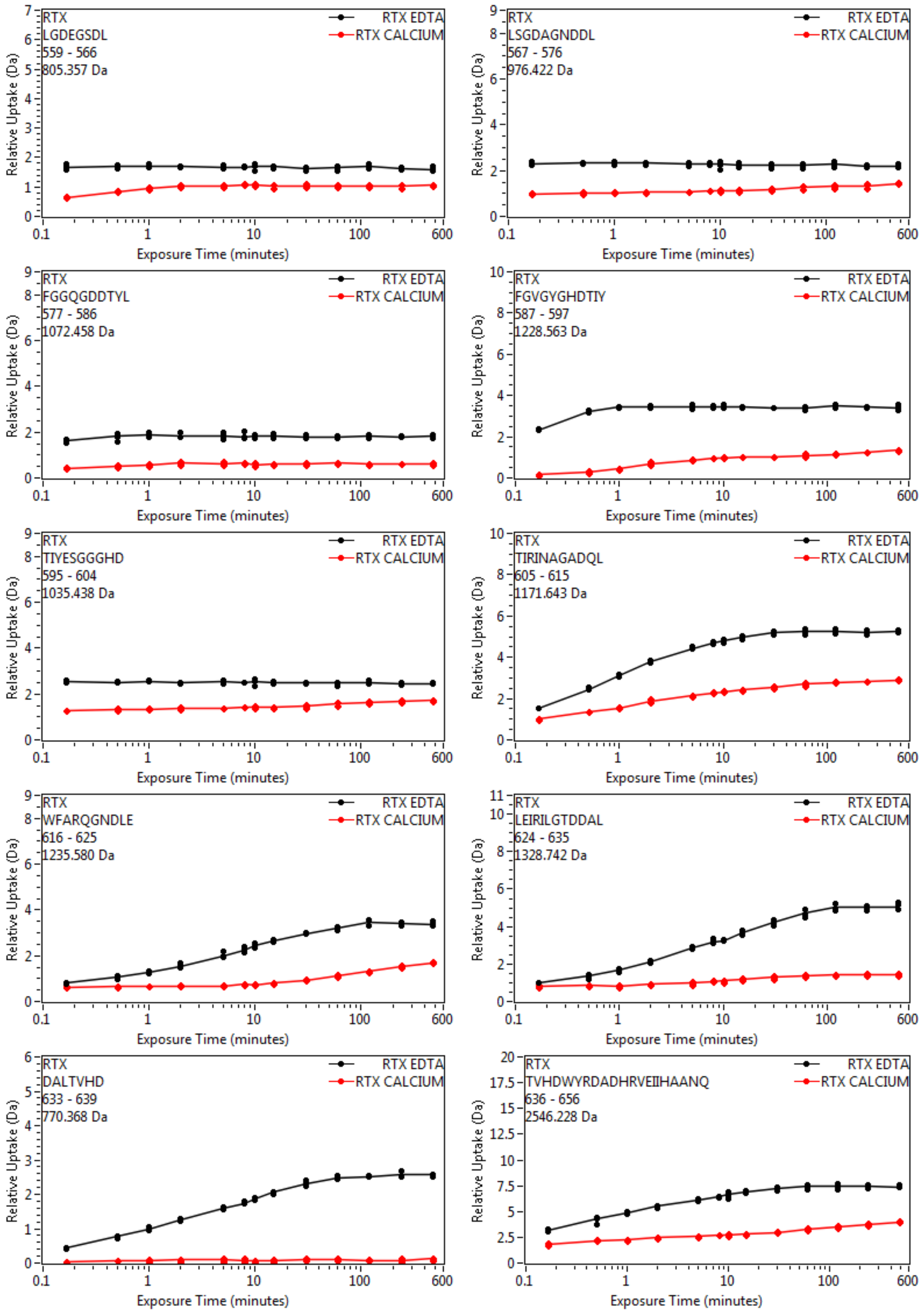

S8. (continued). HDX results for individual RD peptides across all conditions.

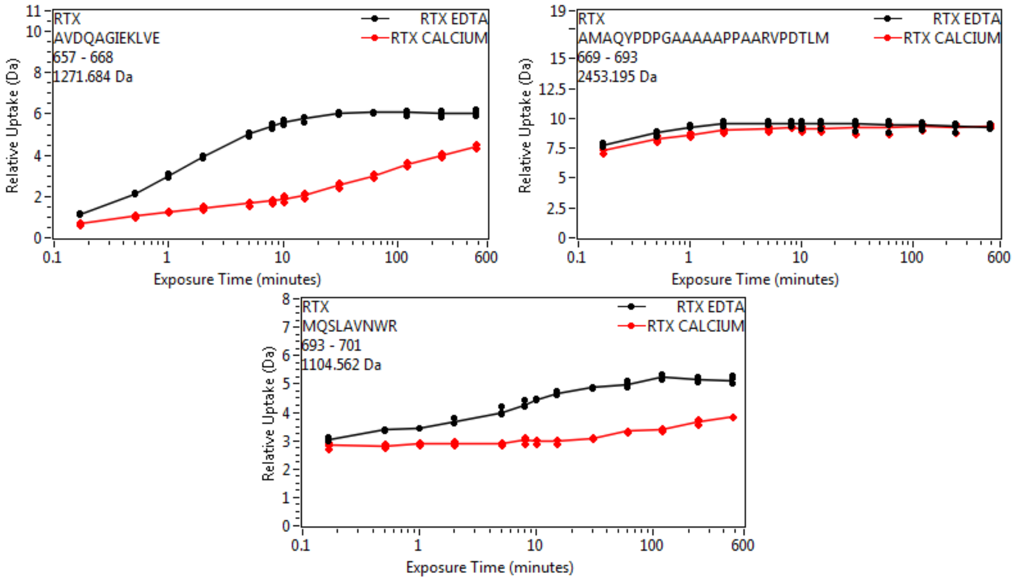

S8. (continued). HDX results for individual RD peptides across all conditions.

Supplementary Tables

Table S1. SAXS data collection and scattering derived parameters.

Data collection parameters		
Instrument	Beamline SWING (synchrotron SOLEIL)	
detector	CCD-based AVIEX	
Sample to detector distance	1.81 m	
Beam geometry	0.8 x 0.15 mm	
Wavelength [Å]	1.033	
q-range [$Å^{-1}$]	0.0062 < q < 0.40	
Exposure time [s]	1	
Temperature [K]	288	
	**Apo-RD**	**Holo-RD**
**Structural parameters**		
Molar extinction coefficient [$M^{-1}.cm^{-1}$]	73 340	
c [$g.L^{-1}$]	0.95	0.38
I(0) Guinier [cm^{-1}]	0.0588	0.0253
R_g Guinier [Å]	82.3±1.0	43.5±0.5
I(0) p(r) [cm^{-1}]	0.0590	0.0254
R_g p(r) [Å]	84.6±1.0	44.7±0.5
D_{Max} [Å]	330	155
**Molecular mass determination**		
$MM_{sequence}$ [kDa]	72.6	72.6
Partial specific volume [$cm^3.g^{-1}$]	0.713	0.713
$MM_{I(0)/c}$ [kDa]	70.5	75.9
$MM_{SAXS\ MoW}$ [kDa]	NA	69.4
**Parameters of the statistical chain model of apo-RD**		
b [Å]	77±5	NA
L [Å]	695±50	NA

Table S2. Populations (accuracy ±5%) of secondary structure elements as determined on the basis of Raman data obtained at T=20°C in aqueous solution.[a]

	Apo-RD		Holo-RD		Structural element
T=20°C	wavenumber	%	wavenumber	%	
**Amide I**	1686 cm^{-1}	25	1689 cm^{-1}	14	Random
(*Amide I′*)	*(1675)*	*(24)*	*(1687)*	*(10)*	
	1672 cm^{-1}	35	1672 cm^{-1}	32	Turn
	(1660)	*(36)*	*(1670)*	*(30)*	
	1660 cm^{-1}	19	1664 cm^{-1}	38	β-strand
	(1648)	*(20)*	*(1661)*	*(38)*	
	1644 cm^{-1}	21	1644	16	Turn
	(1631)	*(19)*	*(1642)*	*(22)*	
	Total Turn	56		48	
		(56)		*(52)*	
	Total β-strand	19		38	
		(20)		*(38)*	
	Total Random	25		14	
		(24)		*(10)*	
	Sum of areas	*100*		*100*	
		(100)		*(100)*	
**Amide III**	1301 cm^{-1}	16	1302 cm^{-1}	14	Turn
	1286 cm^{-1}	13	1287 cm^{-1}	16	Turn
	1267 cm^{-1}	25	1265 cm^{-1}	18	Random
	1248 cm^{-1}	26	1249 cm^{-1}	16	Turn
	1231 cm^{-1}	20	1231 cm^{-1}	36	β-strand
	Total Turn	55		46	
	Total β-strand	20		36	
	Total Random	25		18	
	Sum of areas	*100*		*100*	

**Mean values**[b]	**Apo RD**		**Holo RD**	
	**mean**	**sd**	**mean**	**sd**
Turn	55.7	0.47	48.7	2.49
β-strand	19.7	0.47	37.3	0.94
Random	24.7	0.47	14.0	3.26

[a]All measurements refer to the solution spectra recorded at 5 g/L RD concentration.

[b]Mean values and standard deviations are calculated on the basis of populations obtained from amide I, amide I′ and amide III.

Table S3. Populations (accuracy ±5%) of different secondary structural elements as determined on the basis of Raman data obtained at T=40°C in aqueous solution.[a]

T=40°C[b]	Apo-RD wavenumber	%	Holo-RD wavenumber	%	Structural element
**Amide I**	1686 cm^{-1}	30	1692 cm^{-1}	16	Random
	1671 cm^{-1}	46	1672 cm^{-1}	34	Turn
	1660 cm^{-1}	-	1664 cm^{-1}	39	β-strand
	1650 cm^{-1}	24	1646	11	Turn
	Total Turn	70		45	
	Total β-strand	-		39	
	Total Random	30		16	
	Sum of areas	*100*		*100*	
**Amide III**	1300 cm^{-1}	15	1301 cm^{-1}	13	Turn
	1284 cm^{-1}	17	1287 cm^{-1}	15	Turn
	1264 cm^{-1}	26	1265 cm^{-1}	18	Random
	1243 cm^{-1}	35	1248 cm^{-1}	17	Turn
	1230 cm^{-1}	7	1231 cm^{-1}	37	β-strand
	Total Turn	67		45	
	Total β-strand	7		37	
	Total Random	26		18	
	Sum of areas	*100*		*100*	

[a]All measurements refer to the solution spectra recorded at 5 g/L RD concentration. [b]Increasing temperature up to 50°C does not induce significant changes in populations.

Table S4. Intensity ratios of the integrated areas (from band decomposition of the 700-925 and 1050-975 cm^{-1} spectral regions) calculated for some characteristic Raman bands from Y and W side chains (T=20°C).

	**Tyrosine (Y) wavenumber (cm^{-1})**		**I_{850}/I_{830}**
Apo-RD	855	830	1,15
Holo-RD	857	833	0,78

	**Tryptophan (W) wavenumber (cm^{-1})**			**I_{760}/I_{1010}**	**I_{760}/I_{877}**
Apo-RD	1011	877	757	3,85	4,10
Holo-RD	1010	877	760	1,03	1,67

Supplementary references

1 Bauche, C. *et al.* Structural and functional characterization of an essential RTX subdomain of Bordetella pertussis adenylate cyclase toxin. *J Biol Chem* **281**, 16914-16926 (2006).

2 Chenal, A., Guijarro, J. I., Raynal, B., Delepierre, M. & Ladant, D. RTX calcium binding motifs are intrinsically disordered in the absence of calcium: implication for protein secretion. *J Biol Chem* **284**, 1781-1789 (2009).

3 Chenal, A. *et al.* Calcium-induced folding and stabilization of the intrinsically disordered RTX domain of the CyaA toxin. *Biophys J* **99**, 3744-3753, doi:10.1016/j.bpj.2010.10.016 (2010).

4 Bejerano, M., Nisan, I., Ludwig, A., Goebel, W. & Hanski, E. Characterization of the C-terminal domain essential for toxic activity of adenylate cyclase toxin. *Molecular microbiology* **31**, 381-392 (1999).

5 David, G. & Pérez, J. Combined sampler robot and high-performance liquid chromatography: a fully automated system for biological small-angle X-ray scattering experiments at the Synchrotron SOLEIL SWING beamline. *J. Appl. Cryst.* **42**, 892-900. (2009).

6 Konarev, P. V., Volkov, V. V., Sokolova, A. V., Koch, M. H. J. & Svergun, D. I. PRIMUS - a Windows-PC based system for small-angle scattering data analysis. *J. Appl. Crystallogr.* **36**, 1277-1282 (2003).

7 Guinier, A. Diffraction of X-rays of very small angles – application to the study of ultramicroscopic phenomenon. *Ann. Phys.* **12**, 161–237. (1939).

8 Svergun, D. I. Determination of the Regularization Parameter in Indirect - Transform Methods Using Perceptual Criteria. *J. Appl. Cryst.* **25**, 495-503 (1992).

9 Fischer, H., de Oliveira Neto, M., Napolitano, H. B., Polikarpov, I. & Craievich, A. F. Determination of the molecular weight of proteins in solution from a single small-angle X-ray scattering measurement on a relative scale. *J. Appl. Cryst.* **43**, 101–109. (2010).

10 Petoukhov, M. V. *et al.* Reconstruction of quaternary structure from X-ray scattering by equilibrium mixtures of biological macromolecules. *Biochemistry* **52**, 6844-6855, doi:10.1021/bi400731u (2013).

11 Bernado, P., Mylonas, E., Petoukhov, M. V., Blackledge, M. & Svergun, D. I. Structural characterization of flexible proteins using small-angle X-ray scattering. *Journal of the American Chemical Society* **129**, 5656-5664, doi:10.1021/ja069124n (2007).

12 Tria, G., Mertens, H. D. T., Kachala, M. & Svergun, D. I. Advanced ensemble modelling of flexible macromolecules using X-ray solution scattering. *IUCrJ* **2**, 207-217. (2015).

13 Moore, B. L., Kelley, L. A., Barber, J., Murray, J. W. & MacDonald, J. T. High-quality protein backbone reconstruction from alpha carbons using Gaussian mixture models. *J Comput Chem* **34**, 1881-1889, doi:10.1002/jcc.23330 (2013).

14 Krivov, G. G., Shapovalov, M. V. & Dunbrack, R. L., Jr. Improved prediction of protein side-chain conformations with SCWRL4. *Proteins* **77**, 778-795, doi:10.1002/prot.22488 (2009).

15 Svergun, D. I., Barberato, C. & Koch, M. H. J. CRYSOL - a program to evaluate X-ray solution scattering of biological macromolecules from atomic coordinates. *J. Appl. Crystallogr.* **28**, 768-773 (1995).

16 Kelley, L. A. & Sternberg, M. J. Protein structure prediction on the Web: a case study using the Phyre server. *Nature protocols* **4**, 363-371, doi:10.1038/nprot.2009.2 (2009).
17 Sotomayor-Perez, A. C., Ladant, D. & Chenal, A. Disorder-to-order transition in the CyaA toxin RTX domain: implications for toxin secretion. *Toxins (Basel)* **7**, 1-20, doi:10.3390/toxins7010001 (2015).
18 Petoukhov, M. V. & Svergun, D. I. Global rigid body modeling of macromolecular complexes against small-angle scattering data. *Biophys J* **89**, 1237-1250, doi:10.1529/biophysj.105.064154 (2005).
19 Siamwiza, M. N. *et al.* Interpretation of the doublet at 850 and 830 cm-1 in the Raman spectra of tyrosyl residues in proteins and certain model compounds. *Biochemistry* **14**, 4870-4876 (1975).
20 Benevides, J. M., Overman, S. A. & Thomas, G. J., Jr. Raman spectroscopy of proteins. *Curr Protoc Protein Sci* **Chapter 17**, Unit 17 18, doi:10.1002/0471140864.ps1708s33 (2004).
21 Miura, T., Takeuchi, H. & Harada, I. Raman spectroscopic characterization of tryptophan side chains in lysozyme bound to inhibitors: role of the hydrophobic box in the enzymatic function. *Biochemistry* **30**, 6074-6080 (1991).
22 Miura, T., Takeuchi, H. & Harada, I. Characterization of individual tryptophan side chains in proteins using Raman spectroscopy and hydrogen-deuterium exchange kinetics. *Biochemistry* **27**, 88-94 (1988).
23 van Grondelle, W. *et al.* Lamination and spherulite-like compaction of a hormone's native amyloid-like nanofibrils: spectroscopic insights into key interactions. *Faraday Discuss* **166**, 163-180 (2013).
24 Englander, S. W. & Kallenbach, N. R. Hydrogen exchange and structural dynamics of proteins and nucleic acids. *Q Rev Biophys* **16**, 521-655 (1983).
25 Sotomayor Perez, A. C. *et al.* Characterization of the regions involved in the calcium-induced folding of the intrinsically disordered RTX motifs from the bordetella pertussis adenylate cyclase toxin. *Journal of molecular biology* **397**, 534-549, doi:S0022-2836(10)00077-X [pii] 10.1016/j.jmb.2010.01.031 (2010).
26 Sotomayor-Perez, A. C., Ladant, D. & Chenal, A. Calcium-induced folding of intrinsically disordered repeat-in-toxin (RTX) motifs via changes of protein charges and oligomerization states. *J Biol Chem* **286**, 16997-17004, doi:M110.210393 [pii] 10.1074/jbc.M110.210393 (2011).
